# Studying attention to IPCC climate change maps with mobile eye-tracking

**DOI:** 10.1371/journal.pone.0316909

**Published:** 2025-01-10

**Authors:** Doga Gulhan, Bahador Bahrami, Ophelia Deroy

**Affiliations:** 1 Department of Psychology, Royal Holloway, University of London, London, United Kingdom; 2 Faculty of General Psychology and Education, Ludwig Maximilian University of Munich, München, Germany; 3 Faculty of Philosophy, Philosophy of Science and the Study of Religion, Ludwig Maximilian University of Munich, München, Germany; CNRS: Centre National de la Recherche Scientifique, FRANCE

## Abstract

Many visualisations used in the climate communication field aim to present the scientific models of climate change to the public. However, relatively little research has been conducted on how such data are visually processed, particularly from a behavioural science perspective. This study examines trends in visual attention to climate change predictions in world maps using mobile eye-tracking while participants engage with the visualisations. Our primary aim is to assess engagement with the maps, as indicated by gaze metrics. Secondary analyses assess whether social context (as social viewing compared to solitary viewing) affects these trends, the relationship between projection types and visual attention, compare gaze metrics between scientific map and artwork viewing, and explore correlations between self-reported climate anxiety scores and attention patterns. We employed wearable, head-mounted eye-tracking to collect data in relatively naturalistic conditions, aiming to enhance ecological validity. In this research, participants engaged with ten world maps displaying near- and far-term climate projections across five data categories, adapted from the online interactive atlas provided by the International Panel on Climate Change (IPCC). To compare scientific information processing with aesthetic perception, participants also viewed two large-scale artworks. Responses to the Climate Change Anxiety Scale (CCAS) were also collected. Participants viewed the displays alone (single-viewing condition, N = 35) or together with a partner (paired-viewing condition, N = 12). Results revealed that the upper parts of the maps, particularly the continental Europe, received significant attention, suggesting a Euro-centric bias in viewing patterns. Spatial gaze patterns were similar between single and paired conditions, indicating that the visual attributes of the maps predominantly shaped attention locations. Although dwell times were comparable, the paired condition showed higher fixation counts, shorter average fixation durations, and longer scanpaths, suggesting a potentially dissociable viewing strategy and more exploratory viewing patterns influenced by social interaction. No substantial differences were observed in attention across projection timeframes or types, although individual variations were noted. Artwork viewing exhibited notably shorter average fixation durations compared to climate map viewing, potentially reflecting different visual engagement styles. Despite positive linear correlations among the four CCAS subscales, there was no apparent correlation between CCAS scores and main gaze metrics, indicating a lack of a direct relationship between self-reported anxiety and gaze behaviour. In summary, visual attention to climate change visualisations appears to be mainly influenced by the inherent visual attributes of the maps, but the social context may subtly influence visual attention. Additionally, the comparison with aesthetic viewing highlights relatively distinct attentional patterns in scientific versus aesthetic engagements.

## 1. Introduction

Most of us have seen maps of the earth, often coloured in shades of red and dark orange, illustrating the predicted rise in temperature or rainfalls over the coming decades. But beyond the broad message that we probably already knew, what did we pay attention to? Maps are a major instrument for reporting and communicating climate change to journalists, politicians, and the wider public [1]. They can convey a rich wealth of the spatial, temporal, and quantitative information agreed upon by the community in a simpler and more vivid manner [2]. Maps make up more than 25% of the visualisations in the annual reports of the International Panel on Climate Change (IPCC). These often global representations are widely circulated and used for further decisions and communication campaigns. The IPCC has invested efforts into building better maps and visualisations [3], although suggestions from researchers for more solution-oriented framing [4] remain valid criticism. Nevertheless, intuitions of experts on what constitutes good design in data visualisation do not always materialise, making it important to test data visualisations with the public empirically.

While map-viewing in general is being explored in various contexts through eye-tracking, aiming to answer a wide range of research questions [5–12], we know little about how people specifically look at world maps, particularly those visualising climate change. This gap can be surprising given how much the behavioural sciences have contributed to climate science communication by measuring public perceptions and attitudes toward the crisis, and how many recommendations for improving visualisations they have made over the years [1, 13, 14], along with improving textual contents [15]. While a few studies have recently confirmed the effectiveness of climate communication with maps [16], other studies raise concerns about possible biases induced by the same visualisations, for instance through the misuse of colour [17, 18]. In parallel, research on artwork perception highlights the importance of viewing context (such as laboratory versus gallery or museum environments, spatial layouts, and the authenticity of artworks) in shaping engagement and judgment, with distinct patterns emerging between categories such as digital versus physical or genuine versus replica [19–26]. However, comparisons between aesthetic and scientific viewing contexts remain underexplored, offering a promising direction for further investigation.

To assess public responses and provide evidence-based recommendations, it is essential to move to flexible and varied data collection methodologies beyond traditional questionnaires and focus groups. To address this, we demonstrate the feasibility and relevance of collecting behavioural data from the public as they engage with climate communications and, briefly, with reproduction of artworks, in situ. Mobile eye-tracking technology provides a relatively objective and unobtrusive means to measure where viewers direct their attention when engaging with visual stimuli. It is adaptable for use on screens, mobile devices, and in virtual reality, and can be scaled up to widespread, in situ implementation.

Our study primarily aimed to deploy and validate mobile eye-tracking in a controlled lab setting to investigate how individuals direct their visual attention to climate projection maps. Secondarily, the study also explored potential differences in visual engagement with scientific versus aesthetic stimuli, providing preliminary insights into how people process information-oriented maps compared to visually expressive artworks, while recognising that engagement in lab-based engagement may differ from that in museum environments. While eye-tracking captures gaze in an agnostic way, it is often assumed that distinct pre-attentive (commonly associated with bottom-up processes) and attentive (commonly associated with top-down processes) mechanisms exist. Arguably, our research primarily focuses on bottom-up responses, where participants’ gaze is likely driven by factors such as the visual saliency of the maps, reflecting early, pre-attentive processing, but also influenced by top-down factors like prior knowledge on the issue. To approximate the viewing conditions that the public may encounter in museums or classrooms, we compared solitary viewing conditions to social conditions, where two individuals looked at the same item at the same time. Here, we tested climate change maps as projected data from the IPCC, and included two artworks, to examine differences between viewing patterns aimed at gathering information versus aesthetic appreciation. We aimed to evaluate the utility of mobile eye-tracking as a tool for collecting data on a large scale outside of lab settings, such as during exhibitions, public events, and in educational settings. Ultimately, understanding public behavioural responses, such as visual attention to climate change visualisations, can yield insights that enhance design strategies, making visualisations more explanatory and inclusive.

## 2. Methods

### 2.1 Participants

This study initially recruited 50 participants through convenience sampling. Three recordings were excluded because the gaze mapping algorithm failed to normalise fixation data, and three additional participants did not provide questionnaire responses. The final sample included 47 participants for gaze analysis and 44 for questionnaire responses (33 females, 8 males, 1 non-binary, 2 unspecified; M_Age_ = 20.93 years, SD_Age_ = 4.14 years, R_Age_ = 18–37 years). Participants, primarily students and staff from Royal Holloway, University of London, were recruited via online platforms and campus flyers.

All participants provided written informed consent. The research protocols were certified by the researcher in accordance with the self-certification guidelines provided by the Research Ethics Committee at Royal Holloway, University of London (approval ID: 3527-TFJT002, 2022-12-02). The study was conducted in compliance with the ethical standards outlined in the Declaration of Helsinki. The recruitment period spanned from 05/12/2022 to 05/03/2023.

Corrective lens inserts were provided for participants requiring glasses where possible, but data from those with high prescriptions or other unusable recordings were excluded. Participants were assigned to either single-viewing (N = 35) or paired-viewing conditions (N = 12, six pairs). Unequal group sizes reflected the practical challenges of recruiting pairs, resulting in more participants in the single-viewing condition. Participants received £5 or course credit as compensation.

### 2.2 Stimuli and materials

This study utilised two sets of stimuli (maps and artworks), divided into two sequentially conducted parts. The experiment was conducted in the available space of the Virtual Reality (VR) Lab at the Department of Psychology, Royal Holloway, University of London (although participants did not use VR). The primary stimuli for Part 1 consisted of ten world maps displayed on a 17-inch laptop monitor (Dell Alienware 2019), depicting global climate change projections, including near and far future scenarios for five key measures: mean temperature, sea surface temperature, sea level rise, anthropogenic CO_2_ emissions, and atmospheric particulate matter concentrations (PM_2.5_). These maps were generated using data visualisation tools [27, 28], provided by the Intergovernmental Panel on Climate Change (IPCC) and were accessible at interactive-atlas.ipcc.ch at the time of writing (see S1 Fig for an overview). The supplementary stimuli for Part 2 included two large-scale artworks printed on A0-sized posters and mounted on the laboratory wall (see S2 Fig for details).

The Climate Change Anxiety Scale (CCAS), a 22-item questionnaire [29], was used to measure participants’ responses to climate change on a 5-point Likert scale, covering four subcategories (see S1 File for the full scale). An exit-questionnaire was also administered to gather optional demographics data and participant feedback.

Gaze data were recorded with the Pupil Invisible mobile eye-tracker using Pupil Invisible Companion App (version 1.4.21). The raw gaze data were pre-processed on the GDPR-compliant Pupil Cloud platform. The maps were presented using PsychoPy (version 2022.2.5), the CCAS and exit-questionnaire were presented using Google Forms (see S3 Fig for the experimental setup).

### 2.3 Design

This study was primarily exploratory and descriptive, aiming to analyse participants’ viewing patterns using mobile eye-tracking data while they engaged with ten world maps depicting the climate crisis and two artworks in a laboratory setting.

Data were collected under two viewing conditions: a primary individual viewing condition, where participants viewed the stimuli alone, and a secondary paired viewing condition, where pairs of participants viewed the stimuli together. Despite challenges in recruiting pairs, which resulted in unequal group sizes (35 and 12 in single- and paired-viewing conditions) the setup allowed for the collection and comparative analysis of gaze metrics across different viewing contexts.

The primary analyses focused on fixation-based metrics and their derivatives, which are often linked to attentional processes, such as overt attention and visual attention guidance. Descriptive statistics such as averages, frequencies, and heatmap visualisations were used to present data rather than formal hypothesis testing, highlighting engagement patterns with the stimuli.

Additionally, the study served as a preliminary evaluation of the feasibility of the research procedures and the analysis pipeline in a laboratory setting and beyond, establishing groundwork for future in-situ experiments in public spaces. A significant goal was to compile a sizeable eye-tracking dataset, which, due to the inherent limitations of mobile eye-tracking systems, was expected to be noisier than data from stationary systems. Lastly, this open dataset and code were prepared for reuse in future research, allowing for expanded analyses.

### 2.4 Procedure

The experiment was conducted during regular working hours. Participants began by receiving detailed written and oral instructions, and the experimenter addressed any questions. They provided written informed consent before being equipped with the mobile eye tracker, with correction lens inserts provided when needed. Although the mobile eye tracker was calibration-free and self-correcting, calibration was visually checked using a standard five-point calibration panel, and offset corrections were applied if necessary.

For the first part of the experiment (map viewing), participants’ calibration was confirmed before the recording started. Each participant was assigned a randomly generated three-digit ID and viewed maps displayed on a laptop in a semi-randomised order, spending at least 30 seconds per map. The near-term projection map of each type was always displayed before the corresponding long-term projection map, but the order of projection types was randomised across participants (see S4 Fig for the procedure diagram). Participants in the paired-viewing condition were encouraged to discuss the maps with their partner, while those in the single-viewing condition viewed them independently.

For the second part (artwork viewing), participants viewed two large-scale paintings mounted on the wall. They carried the companion device with them and were free to choose the viewing order and spend as much time as they wished on the artworks, moving freely around them.

After both sessions, participants filled out the 22-item climate change anxiety scale and optionally provided demographic information and comments. The experiment concluded with a debriefing by the experimenter.

### 2.5 Data analysis

Data analysis progressed through several stages, from raw recordings to detailed analyses. Recordings from the companion device were uploaded to Pupil Cloud, a GDPR-compliant online platform for data processing and visualisation. Fixation detection used an extended I-VT (Identification by Velocity Threshold) algorithm. Data quality was inspected informally by visually checking raw gaze data overlaid with fixations on Pupil Cloud, alongside fixation duration rates relative to recording duration. No valid recordings were excluded. Some spatiotemporal random noise in gaze data, as reported by the manufacturer, was assumed not to affect the primarily descriptive analyses.

To map the XY-coordinates of raw gaze and fixation data, two streams of parallel processing were employed on the cloud. This dual approach was designed to take advantage of a newly available algorithm in beta version at the time of data collection, which was later released as a stable version. The first, marker mapper enrichment (MME), utilised fiducial markers (Apriltags) placed around the laptop monitor and paintings to define areas of interest (AOIs). Recordings were manually time-stamped for segmentation, and markers served as anchor points to normalise gaze data. The second, reference image mapper (RIM), employed video recordings and snapshots to create a structure-from-motion model for normalisation. While both methods produced comparable data structures, RIM demonstrated higher accuracy and was used for all subsequent analyses (see S5 Fig for an overview). RIM preserved the height-to-width ratio of stimuli in pixel-based values, unlike MME, which distorted the aspect ratio during normalisation. Surface-normalised fixation data were used to create heatmaps visualising viewing patterns.

The enriched dataset, along with supplementary materials, was uploaded to Kaggle and Google Colab for further analysis using Python-based notebooks (e.g., pandas, matplotlib, seaborn), alongside offline software (e.g., jamovi). Affinity Designer and Affinity Photo were used to refine plot outputs. Data and analyses were also uploaded to the Open Science Framework (osf.io). Primary gaze metrics included total fixation duration (in milliseconds and percentage), fixation count, average fixation duration, and proxy saccadic scanpath length. As mobile eye tracking lacked constant participant-to-stimulus distance, scanpath length was calculated using pixel-based on-screen values as a proxy.

## 3. Results

The results section generally reports descriptive values, specifically the mean (M) and standard error of the mean (±SEM), unless stated otherwise. The primary indicators of participant engagement with the maps and artworks were derived from fixation-based metrics, including total fixation duration (also referred to as dwell time), fixation count, average fixation duration, and proxy saccadic scanpath length. Instances of transient engagement, such as brief glances at fiducial markers, were excluded from the analysis due to their minimal duration. As previously described in the Data Analysis section, the pre-processed, enriched data were obtained using the reference image mapper (RIM) technique.

### 3.1 Descriptive statistics for maps

The main descriptive statistics for the maps reflect averages across all stimuli, without differentiation by map type. Supplementary statistics were broken down either by map projection timeframe or projection type. Sample sizes were 35 for the single viewing condition and 12 for the paired viewing condition, with each of the 10 stimuli viewed under both conditions. This resulted in a total of 350 observations for the single viewing and 120 for the paired viewing conditions.

**Fig 1:**
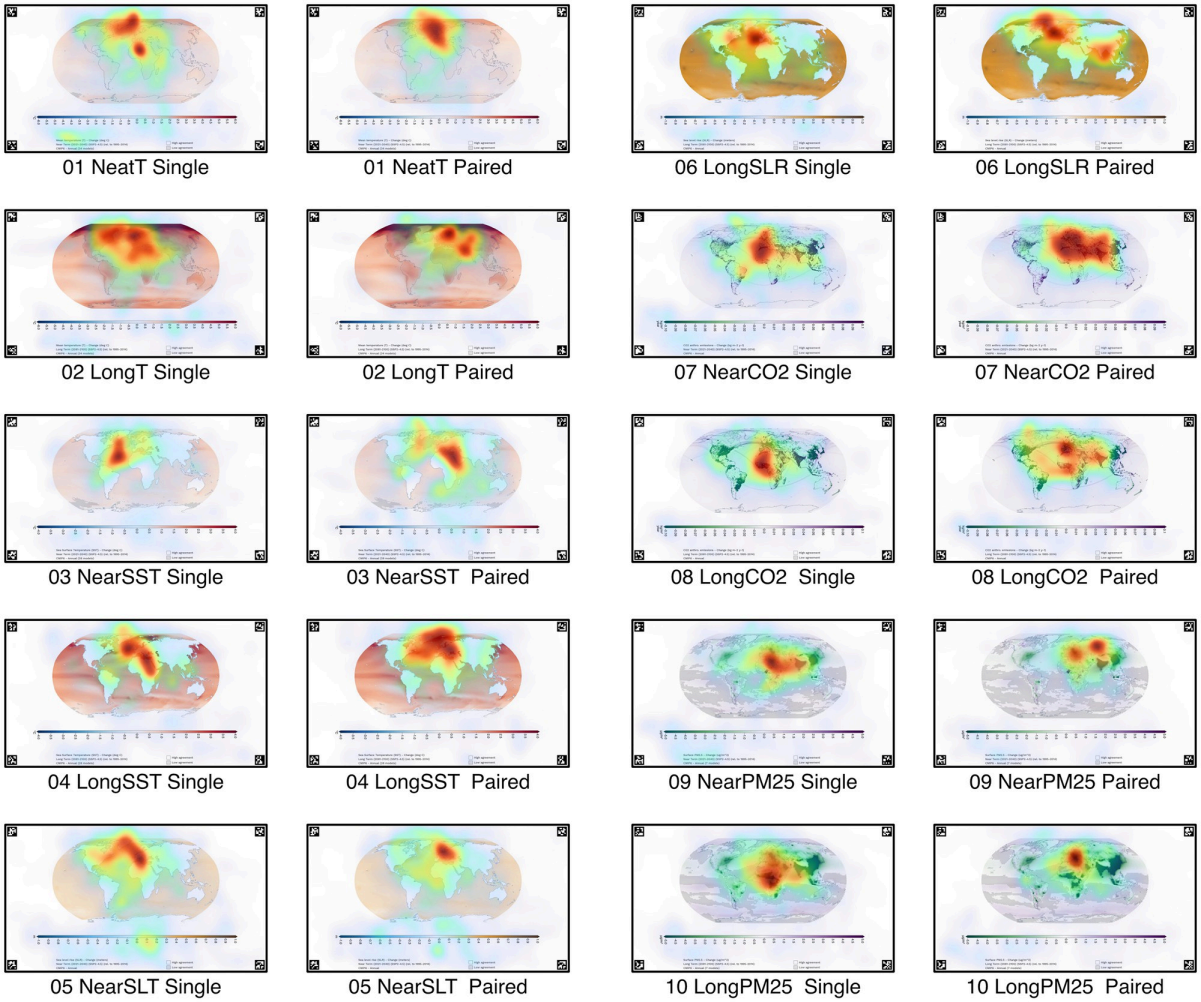
Fixation heatmaps for maps. Heatmaps illustrate fixation distributions across maps under single and paired-viewing conditions, using a green-to-red colour scale for shorter to longer fixation durations. Single- and paired-viewing conditions are shown in the first/third and second/fourth columns, respectively. Spatial common ground generally exhibits an upper-central tendency. Substantial overlap between conditions suggests that image-based saliency predominantly drives visual attention, over social context.

Initial visualisation using cumulative heatmaps revealed a strong upper-central tendency in viewing patterns, indicating that regions on and around continental Europe received significant attention across most cases. These heatmaps also illustrated comparable spatial patterns between single and paired viewing conditions. Notably, a considerable number of fixations were concentrated on the scales and on areas of the maps displaying the minima and maxima values of the corresponding scale. These areas often represent the most salient regions in terms of contrast and colour, suggesting that such bottom-up factors are major determinants of spatial attention location among participants. For an illustrative overview of these patterns, refer to Fig 1, which displays the heatmaps of the maps.

**Fig 2:**
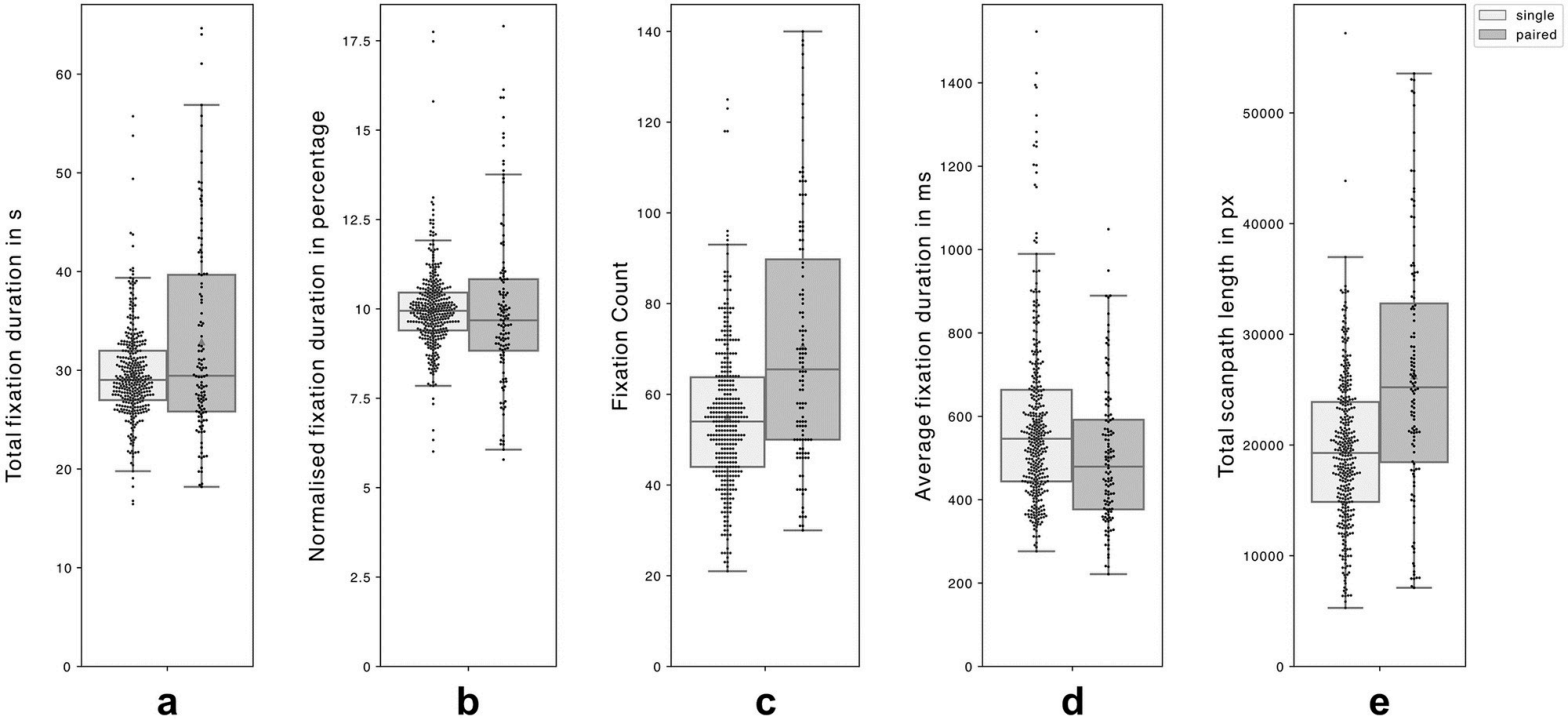
Box plots of gaze metrics for maps. Box plots illustrate five gaze metrics across all maps (single-viewing in light grey and paired-viewing in dark grey): **(a)** total fixation duration (s), **(b)** normalised fixation duration (%), **(c)** fixation count, **(d)** average fixation duration (ms), and **(e)** proxy scanpath length (pixels). Whilst total fixation durations were comparable between conditions, paired viewing showed higher fixation counts, shorter average fixation durations, and longer scanpaths. Each plot displays the range (excluding outliers), interquartile range, median, and mean (triangle overlay).

In comparing single and paired viewing conditions (N_SampleSingle_ = 35, N_StimulusSingle_ = 350 and (N_SamplePaired_ = 12, N_StimulusPaired_ = 120), the total fixation duration, or dwell time, averaged around 30 seconds per map, showing some variations within each condition but relatively minimal variation between conditions: M_Single_ = 29.70 s (±.25), M_Paired_ = 32.92 s (±.94). This represented approximately 10% of the total dwell time across each map for both conditions. The number of fixations, or fixation count, was relatively lower in the single viewing condition compared to the paired viewing condition: M_Single_ = 55.12 (±.87), M_Paired_ = 70.70 (±2.47). In line with this difference in the fixation count, and as a derivative metric to the previous two, the average fixation duration was longer in the single viewing condition compared to the paired viewing condition: M_Single_ = 586.43 ms (±11.29), M_Paired_ = 504.45 ms (±15.29). The proxy scanpath length, measured as the cumulative sum of Euclidean distances between successive fixations based on reference images with 1920 × 1080 pixel dimensions, was shorter in the single viewing condition compared to the paired viewing condition: M_Single_ = 19534.89 px (±365.87), M_Paired_ = 26288.61 px ±1036.95), refer to Fig 2 for an overview as box plots and S1 Table for aggregate metrics.

Despite the relatively low sample size and exploratory nature of the research, a nonparametric ANOVA (Kruskal-Wallis test) was used to analyse these metrics. While fixation duration showed no significant differences, fixation count, average fixation duration, and scanpath length displayed significant differences between single and paired viewing conditions (see S2 Table for detailed breakdown of χ2, df, p, ε^2^ values).

Additionally, descriptive statistics were reported based on two categorisations: map projection timeframe (near and far future) and map projection type (main temperature, sea surface temperature, sea level rise, anthropogenic CO_2_ emissions, and fine particulate matter PM_2.5_). Metrics were similar between the two timeframes but showed some variation across the five types of projections. The overall variance was generally larger, especially for the maxima, in paired condition for all metrics except average fixation duration. These results are presented in S3 Table. To further illustrate minor trends and detailed data across the five metrics, results were divided by both viewing conditions and the ten stimuli. S6 Fig displays box plots overlaid with individual data points, and S4 Table provides a comprehensive descriptive summary.

Lastly, given the inherent gaze-estimation accuracy limits of the mobile eye tracker (approximately reported as ≈4° by the whitepaper from the manufacturer), conducting a highly granular AOI-based analysis may lead to significant errors: particularly for a generic viewing condition, the monitor surface area might roughly translate to a surface of a 38° × 21.5° of visual angles. For instance, it is impractical to confidently display fixations on individual countries due to these accuracy limitations and the relatively low sample size, which could skew the gaze estimation errors beyond mere random noise in the data. Nevertheless, as a proof of concept, the stimulus was divided into two broad AOIs: the upper section representing the map and the lower section the scale. On average, participants spent four times as much time viewing the main map compared to the scale at the bottom. This 80–20% relative dwell time difference was interestingly consistent across both viewing conditions and remained relatively stable when broken down by individual maps (see S5 Table).

### 3.2 Descriptive statistics for paintings

Similar to the analysis of the maps, descriptive statistics for the two paintings were calculated, averaging across stimuli. Sample sizes were 35 for the single viewing condition and 12 for the paired viewing condition, with each of the two stimuli viewed under both conditions. This resulted in a total of 70 observations for the single viewing and 24 for the paired viewing conditions.

**Fig 3:**
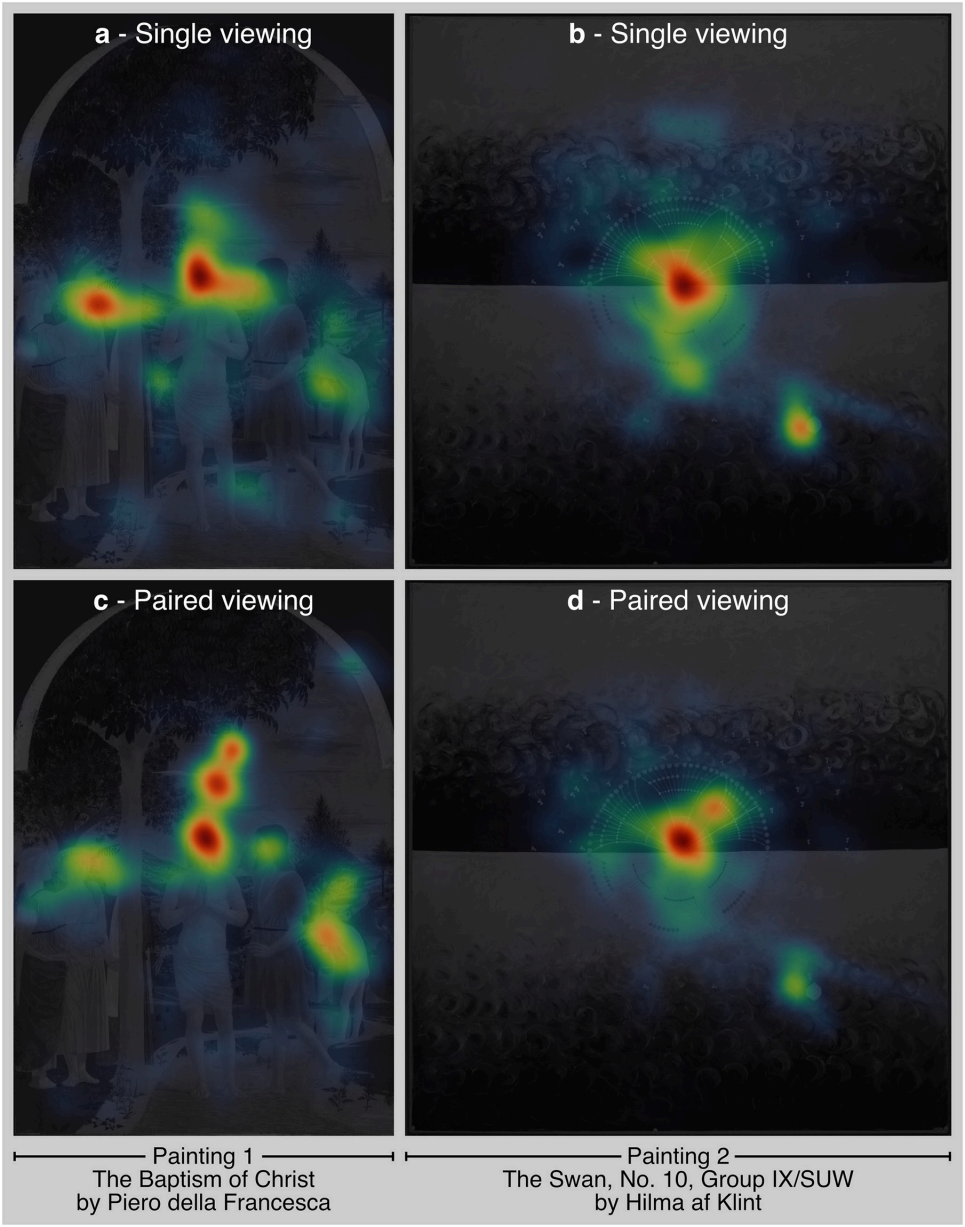
Fixation heatmaps for paintings. Heatmaps show fixation distributions for two paintings, based on data from all participants in both single-viewing **(a-b)** and paired-viewing **(c-d)** conditions.

Initial visualisation using cumulative heatmaps for the paintings indicated a pronounced central tendency consistent with the layout of the artworks. The first painting, being more figurative with numerous elements, exhibited diverse focal points such as faces and bodies, resulting in a more dispersed gaze pattern across both X and Y axes. In contrast, the second painting, which is more abstract and centrally composed, showed gaze dispersion primarily concentrated at the centre and one particular area on bottom-right (albeit gaze dispersions were not further plotted). These differences highlight how compositional elements influence visual attention as indexed by cumulative fixations. For a detailed view of these attentional distributions, refer to Fig 3, showcasing the heatmaps of the paintings.

**Fig 4:**
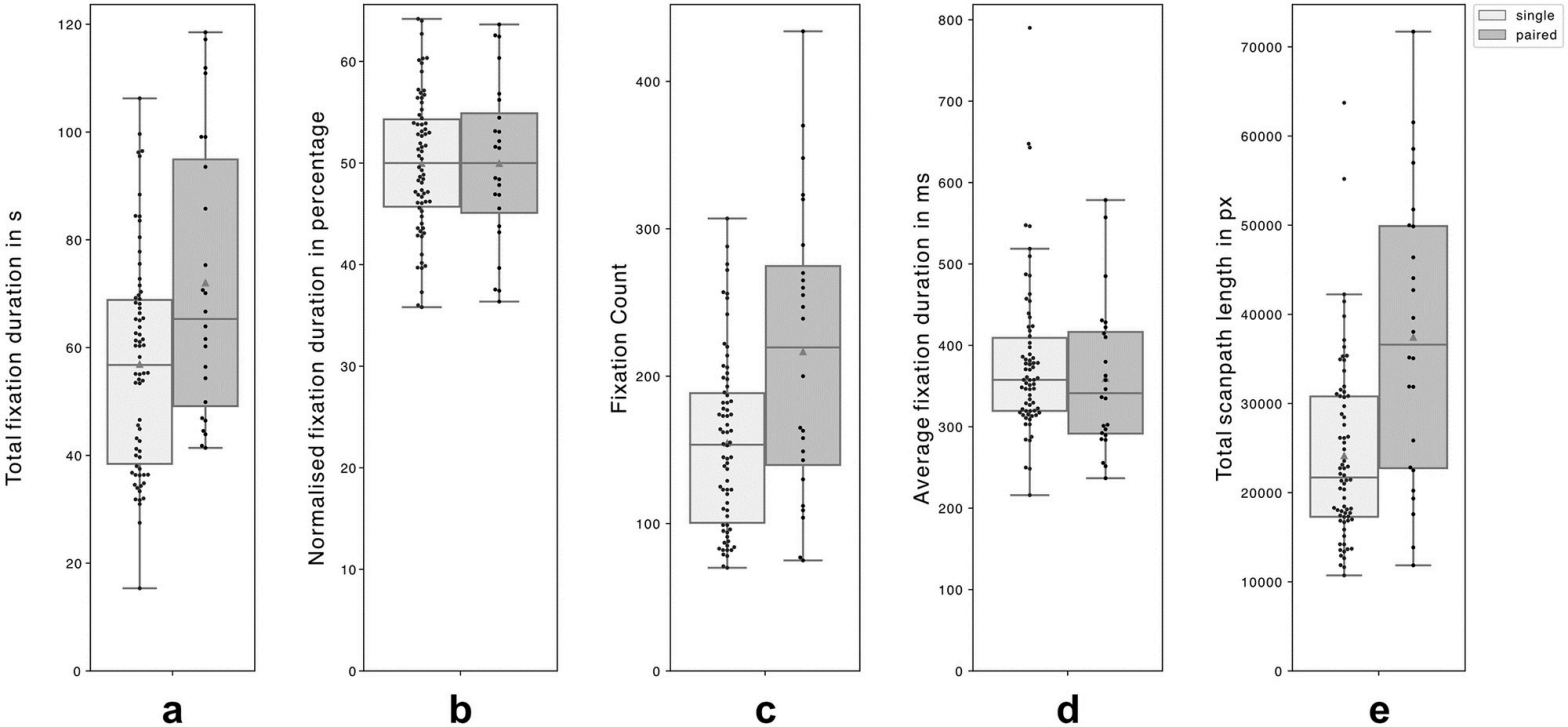
Box plots of gaze metrics for paintings. Box plots depict five gaze metrics for two paintings (single-viewing in light grey and paired-viewing in dark grey): **(a)** total fixation duration (s), **(b)** normalised fixation duration (%), **(c)** fixation count, **(d)** average fixation duration (ms), and **(e)** proxy scanpath length (pixels). On average, single viewing showed slightly shorter dwell times, lower fixation counts, slightly longer average fixation durations, and significantly shorter scanpath lengths. Each plot shows the range (excluding outliers), interquartile range, median, and mean (triangle overlay).

In comparing single and paired viewing conditions (N_SampleSingle_ = 35, N_StimulusSingle_ = 70 and (N_SamplePaired_ = 12, N_StimulusPaired_ = 24), the total fixation duration, or dwell time, averaged about one minute per painting, with some variation within each condition and slightly shorter durations in the single condition compared to the paired: M_Single_ = 56.97 s (±2.40), M_Paired_ = 72.09 s (±5.30). The number of fixations, or fixation count, was relatively lower in the single viewing condition compared to the paired: M_Single_ = 154.87 (±7.07), M_Paired_ = 216.86 (±20.27). Additionally, the average fixation duration was only slightly longer in the single viewing condition: M_Single_ = 380.51 ms (±11.41), M_Paired_ = 359.95 ms (±18.68). The proxy scanpath length, measured as the cumulative sum of Euclidean distances between successive fixations based on reference images (685 × 1000 pixels for the Baptism of Christ (Painting #1) and 983 × 1000 pixels for The Swan (Painting #2)), was significantly shorter in the single viewing condition: M_Single_ = 24196.75 px (±1205.88), M_Paired_ = 37468.70 px (±3332.50), refer to Fig 4 for an overview as box plots and S6 Table for aggregate metrics, divided by viewing two conditions.

Despite the relatively low sample size and the exploratory nature of the research, a nonparametric ANOVA (Kruskal-Wallis test) was employed for statistical analysis. While average fixation duration showed no significant differences, total fixation duration, fixation count, and scanpath length exhibited significant differences across conditions (see S7 Table for a detailed breakdown of χ2, df, p, ε^2^. Similar trends were observed when the data were further disaggregated by the two paintings (see S7 Fig and S8 Table).

Although not subjected to statistical comparison, it may be useful to highlight observed trends between map and painting viewing. On average, participants spent about twice as much time viewing paintings compared to maps, as indexed by dwell time. This discrepancy may be attributed to several factors: Participants might have found artworks more engaging than climate crisis maps, or the smaller number of paintings (two) compared to maps (ten) could have allowed for longer individual viewing times per painting. Additionally, the maps might not have contained as much visual information or complexity, which might have required less time to view. Another notable difference was also observed in average fixation duration, which was highly shorter for painting viewing compared to map viewing. Longer average fixation durations sometimes suggest a higher cognitive load, while shorter fixations might indicate that the task of viewing paintings was less cognitively demanding, or that the information was easier to process. This difference could also be influenced by methodological factors: fixation detection algorithms might perform differently on screen-based stimuli versus in situ observation, with the latter possibly affected by participant mobility during painting viewing. Lastly, in both settings, fixation counts were higher in paired viewing conditions, indicating a consistent trend across this metric. Interestingly, while dwell times were comparable between single and paired map viewing conditions, they differed significantly in painting viewing, suggesting a minor preferential difference between the two types of stimuli.

### 3.3 Climate change anxiety scale (CCAS) responses

Irrespective of the viewing condition, whether single or paired, the analysis of the climate change anxiety scale involved four subscales of the 22-item questionnaire, with responses gathered using a 5-point Likert scale. For a visual representation of the responses, refer to S8 Fig for the frequency plot of individual items, S9 Fig for the aggregated frequency plot across the four subscales, and S9 Table for a statistical breakdown.

Overall, levels of climate change anxiety were relatively low across the sample. For the first two, often highly correlated subscales (cognitive-emotional impairment and functional impairment), participants typically reported low scores. More than half of the responses were “never,” and approximately a quarter were “rarely.” However, a small subset of participants exhibited mid or high scores on these measures, suggesting notable levels of climate-related anxiety for a minority of participants within the group.

Furthermore, responses on the personal experience with climate change subscale, and more distinctly on the behavioural engagement subscale, were comparatively higher. While the distribution of responses on the personal experience subscale was relatively even across all five points of the Likert scale, the behavioural engagement subscale showed a negatively skewed distribution. This suggests that on average, participants either exhibited or aspired to positive behaviours towards addressing the climate crisis.

When investigating the relationships between the four subscales of the climate change anxiety scale (CCAS), the 5-point Likert scale responses were treated as ordinal data, assigning values from 0 (never) to 4 (almost always). Despite the frequency distributions varying across the subscales, their relationships were examined through cross-correlation, using Spearman’s rho and Kendall’s tau-b. These analyses showed positive linear correlations between the subscales, as illustrated in S10 Fig, and detailed in S10 Table. This suggests that a combined CCAS score could be formed as a cohesive construct for further analysis.

To explore the relationship between this aggregated CCAS score (treated as ordinal data) and the four primary gaze metrics (treated as continuous data), we conducted correlation analyses. The results, however, indicated no significant correlations; all relationships were effectively flatlined across the metrics. This lack of significant findings implies that there is no immediate or obvious connection between main gaze metrics and self-reported climate anxiety, as detailed in S11 Table. Given these outcomes, we did not proceed further, such as dividing participants into low and high anxiety groups based on median, quartile, or range-based splits.

## 4. Discussion and conclusions

The present study established the relevance of using mobile eye-tracking to examine how people look at climate change maps, and provided insights for scaling it up to more naturalistic settings, notably social ones. Despite individual differences, on average, the comparison between conditions during map viewing suggests that social interaction can subtly alter gaze patterns. Paired viewing was associated with higher fixation counts, shorter average fixation durations, and longer scanpaths, though dwell times remained comparable. These variations may indicate differences in visual and cognitive processing, such as varying cognitive efforts [30], or social motivation [31].

Attentional hotspots, indexed by fixation heatmaps, were broadly similar, indicating that the content of visual stimuli primarily captures attention, often displaying a Euro-centric bias. This implies that the bottom-up, object-based factors such as image saliency may have a larger effect on viewing patterns. However, dissimilarities can be partially explained by how social contexts shape viewer engagement, thereby influencing their information processing strategies.

Additionally, gaze metrics differed between viewing maps and artworks. Scientific information processing and aesthetic perception showed some distinct viewing strategies, with map viewing generally associated on average with longer fixation durations. These preliminary findings align to some extent with prior work highlighted in the Introduction, suggesting that viewing context (including differences between laboratory and museum environments) and the authenticity of stimuli (such as reproductions versus originals) can influence gaze patterns and overall engagement with visual stimuli. Although not directly comparable (given that this study was conducted in a lab setting with reproductions of artworks), it is worth considering what constitutes the genuine context for climate change maps, which are encountered in a variety of settings.

Although there were significant positive linear cross-correlations among the subscales of the CCAS, the lack of a significant linear correlation between main gaze metrics and climate anxiety scores suggests that visual attention, as indexed by main gaze metrics, may not directly relate to self-reported trait anxiety levels. It is important to note that no additional measures were used to capture contextual emotional responses of participants to the content in this study.

The small size of the paired-viewing group, due to convenience sampling, and the inherent data noise in mobile eye-tracking need to be considered when assessing these pilot results. The imbalance between single- and paired-viewing conditions, as well as the limited diversity in gender and age, further limits the generalisability of the findings. These constraints reflect the challenges of recruiting larger, more balanced samples for mobile eye-tracking studies, which typically require specialised equipment and substantial resources. Additionally, participants might have treated the viewing differently within the experimental setting compared to a naturalistic environment, such as a museum or classroom. Nonetheless, the observed differences between isolated and social settings, and between maps and artworks, show that the method can successfully capture differences in viewing patterns, even under these conditions. This study provides a foundation for future research to consider the impact of various types of climate-related visual stimuli on a broader audience. Extending this research to more ecologically valid settings, as highlighted earlier, could help further clarify how real-world contexts shape engagement with scientific and aesthetic stimuli. Additionally, integrating momentary affective assessments and qualitative assessments in mixed-methods designs could also provide deeper insights into the cognitive and emotional dimensions of viewer engagement.

Our study highlights the potential of mobile eye-tracking for understanding how people engage with climate projections. Behavioural science has been hinted as a precious source of recommendations to enhance climate visualisations in four key areas: how to direct visual attention, reduce visual complexity, support inference-making, and integrate text with graphics [1], and this study clearly demonstrates how it can contribute to the first objective. This should not mean that the other aspects are equally essential and require further investigation. Visual complexity and text-graphic integration can also be tested with eye-tracking and highlights future uses for the methods. The reason not to pursue those here is that our approach aimed to mimic everyday encounters with climate content by allowing participants to simply view images naturally, without additional inference, understanding, or memory questions as in some other research [32].

The differences in visual engagement between single and paired viewing conditions, as well as between maps and artworks, underscore the potential for visual communication strategies better tailored to contexts. Research indicates that changing graph designs derived from IPCC reports can intentionally alter perceptions and even shift the credibility of the presented data [33]. Strategies leveraging different media formats can enhance public understanding of climate science. For instance, the memorability of visualisations [34] can be utilised to promote climate action, with eye-tracking and visual attention serving as useful tools to assess the effectiveness of different visualisations.

Although our study used static images, previous research has shown that interactive visualisations that are more personally relevant yield promising results in terms of perceived reality of climate change, attitude certainty, and concern [35]. Tailoring of communication can also be done for different viewers. In the present case, we did not find evidence of differences between individuals with different trait climate anxiety. Other directions remain open. For instance, individuals with varying levels of optimism, when presented with climate change messages in text, show different allocations of visual attention and recall, underlining attentional bias and suggesting the need to redesign our communications [36]. Preliminary evidence also suggests different viewing strategies and actions between political groups (liberals and conservatives), highlighting the ideological influence on visual attention [37] and the need for tailored communication tools to address such attentional and perceptual biases [38].

Behavioural sciences can play a crucial role in identifying and overcoming psychological barriers to climate action [39]. However, creating effective interventions poses significant challenges, as evidenced by large-scale cross-cultural studies [40]. Therefore, from a methodological standpoint, employing behavioural data collection using mobile, screen-based, or XR eye-tracking can be seen as essential for pinpointing visual attention, and later on help for improving climate communication and interventions.

## Supporting information

S1 Fig Overview of main stimuli, ten climate crisis maps. (PDF)

S2 Fig Overview of supplementary stimuli, two paintings. (PDF)

S3 Fig Data recording setup. (PDF)

S4 Fig Procedure diagram for viewing ten maps. (PDF)

S5 Fig Online data pre-processing using MME and RIM methods. (PDF)

S6 Fig Breakdown of box plots of descriptive statistics for main gaze metrics by map. (PDF)

S7 Fig Breakdown of box plots of descriptive statistics for main gaze metrics by painting. (PDF)

S8 Fig Frequency plot of survey results, item-wise. (PDF)

S9 Fig Frequency plots of survey results, by four subcategories. (PDF)

S10 Fig Correlation plots between four subscales of the CCAS. (PDF)

S1 Table Gaze metrics for maps. (PDF)

S2 Table Nonparametric ANOVA (Kruskal-Wallis test) for maps, comparing single and paired viewing conditions. (PDF)

S3 Table Gaze metrics for maps, broken down by projection timeframe and type. (PDF)

S4 Table Gaze metrics for maps, fully broken down. (PDF)

S5 Table Gaze metrics for maps divided into two AOIs. (PDF)

S6 Table Gaze metrics for paintings. (PDF)

S7 Table Nonparametric ANOVA (Kruskal-Wallis test) for paintings, comparing single and paired viewing conditions. (PDF)

S8 Table Gaze metrics for paintings, fully broken down. (PDF)

S9 Table Survey descriptive statistics by CCAS subscales. (PDF)

S10 Table Survey correlation statistics for CCAS subscales. (PDF)

S11 Table Correlations between gaze metrics and total CCAS score. (PDF)

S1 File The Climate Change Anxiety Scale (CCAS). (PDF)
