# Supplementary material for: Studying attention to IPCC climate change maps with mobile eye-tracking
Source: PLoS One. 2025 Jan 10;20(1):e0316909. doi: 10.1371/journal.pone.0316909 (PMC11723542; doi:10.1371/journal.pone.0316909)
Supplement: S1 Fig — (PDF) [file pone.0316909.s001.pdf]

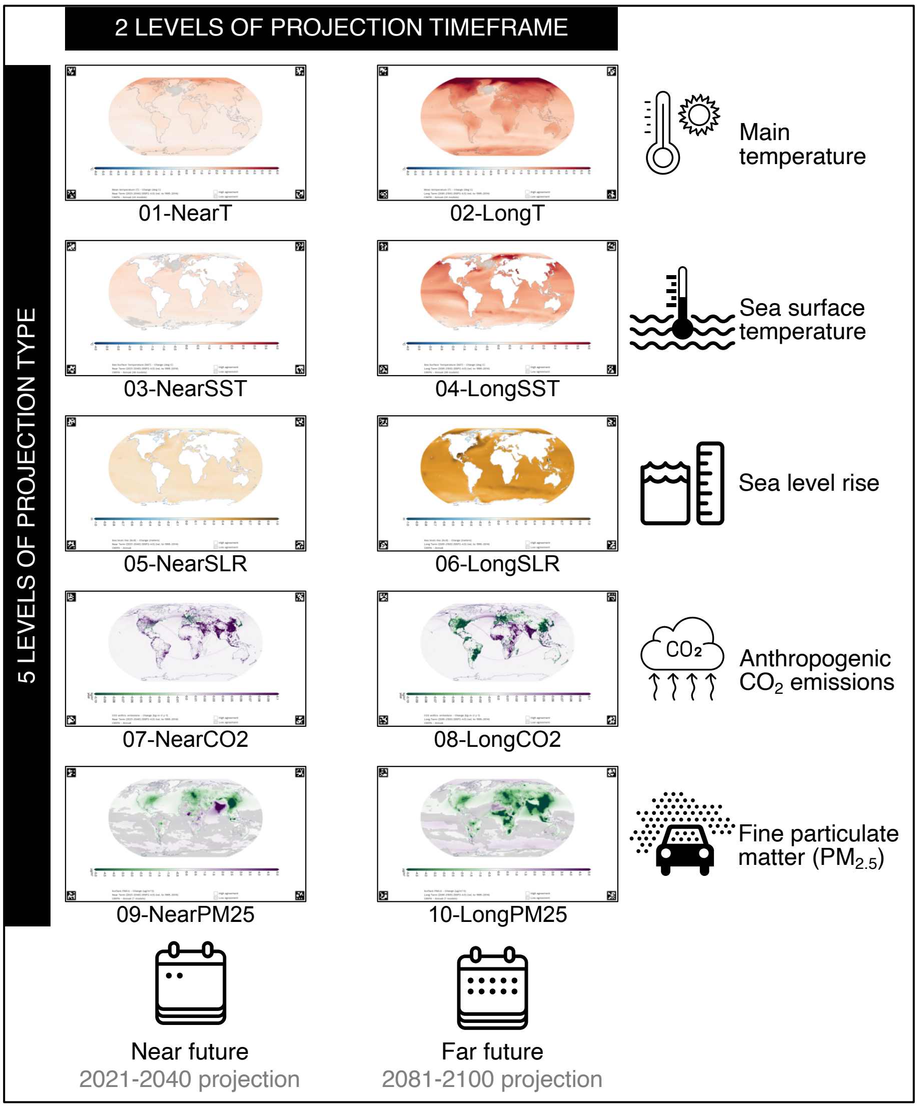

S1 Fig. Overview of main stimuli, ten climate crisis maps.

This figure provides an infographic overview of the main stimuli, consisting of five pairs of world maps with a Robinson projection. These maps were generated using the online data visualisation tool from the Intergovernmental Panel on Climate Change (IPCC) and downloaded from their website (ipcc.ch and interactive-atlas.ipcc.ch). Each map features a heatmap overlay representing a specific type of climate projection, accompanied by a relevant scale and brief data description below it. The maps are organised into two columns representing different projection timeframes: the near future (2021-2040) and the far future (2081-2100), with corresponding icons beneath for illustrative purposes. The five rows depict different projection types: temperature change in °C, sea surface temperature change in °C, sea level rise in metres, anthropogenic CO_2 emissions in kg/m^2 per year, and particulate matter ($PM_{2.5}$) concentration changes in µg/m^3, with icons on the right for illustration. In the experiment, these maps were displayed full-screen on a 17-inch laptop monitor. Each map presentation was preceded by a title card (not shown here) that displayed these icons and titles, informing participants about the content of the upcoming map. A countdown from 30 seconds and a brief instruction text afterwards (not illustrated here) were also present during each trial, indicating that participants could advance to the next map at any time by pressing the spacebar. Below the maps, naming conventions are listed, which are used in subsequent data visualisations like bar plots in this paper. Note that small Apriltags around each map were initially intended for the Marker Mapper (MM) preprocessing pipeline but were not used, as the Reference Image Mapper (RIM) preprocessing pipeline was used instead, which does not require Apriltags. (See Methods for details).
