# Supplementary material for: Studying attention to IPCC climate change maps with mobile eye-tracking
Source: PLoS One. 2025 Jan 10;20(1):e0316909. doi: 10.1371/journal.pone.0316909 (PMC11723542; doi:10.1371/journal.pone.0316909)
Supplement: S2 Fig — (PDF) [file pone.0316909.s002.pdf]

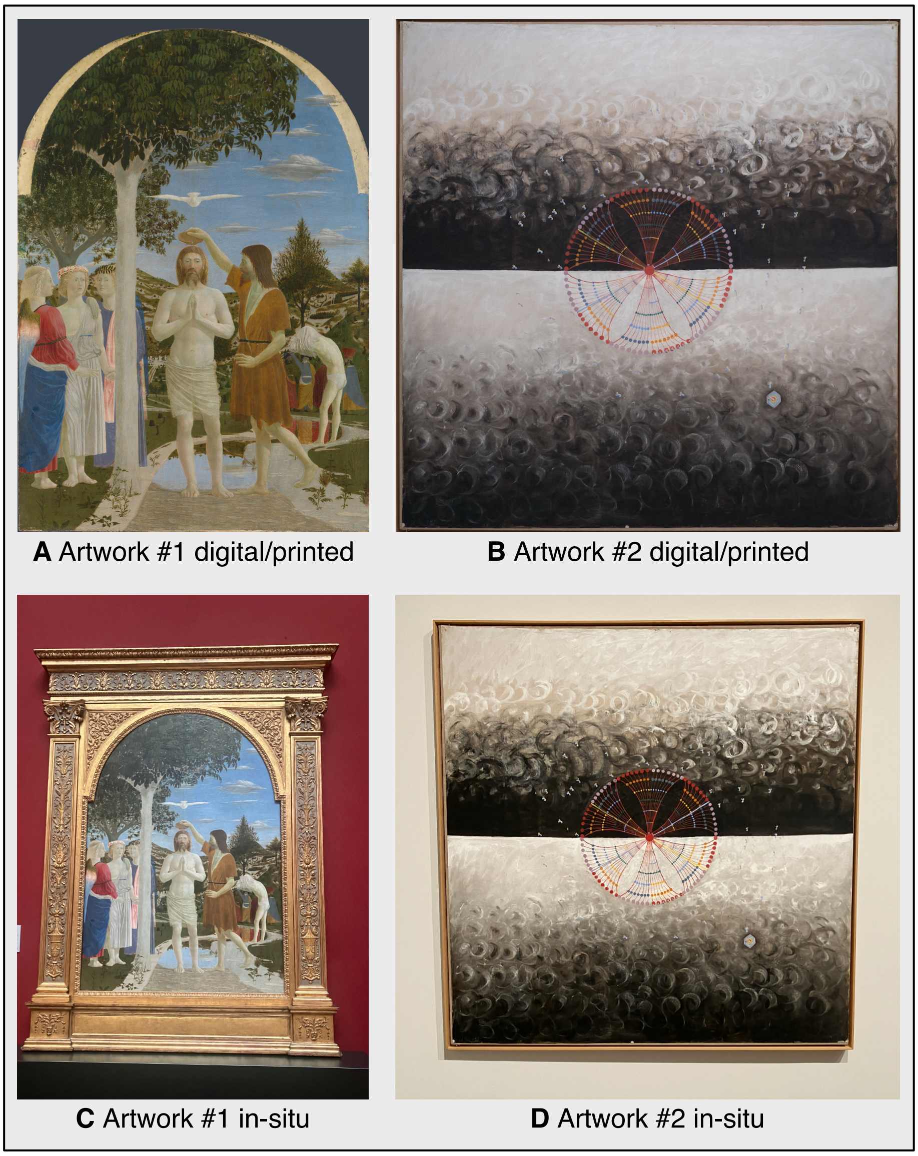

S2 Fig. Overview of supplementary stimuli, two paintings.

This figure showcases high-resolution digital images of two paintings printed on A0-sized posters, serving as a small supplementary stimulus set. These paintings were selected for their distinct content and downloaded from Wikimedia Commons (commons.wikimedia.org). **Top-left (a):** The Baptism of Christ by Piero della Francesca, completed circa 1448–50, actual size approximately 167 cm × 116 cm. **Top-right (b):** The Swan, No. 10, by Hilma af Klint, completed in 1915, actual size approximately 150 cm × 150 cm. **Bottom-left (c)** and **Bottom-right (d)** feature photographs of the original artworks as seen at the National Gallery, London, and Tate Modern, London, respectively, photographed by DG.
