# Supplementary material for: Studying attention to IPCC climate change maps with mobile eye-tracking
Source: PLoS One. 2025 Jan 10;20(1):e0316909. doi: 10.1371/journal.pone.0316909 (PMC11723542; doi:10.1371/journal.pone.0316909)
Supplement: S3 Fig — (PDF) [file pone.0316909.s003.pdf]

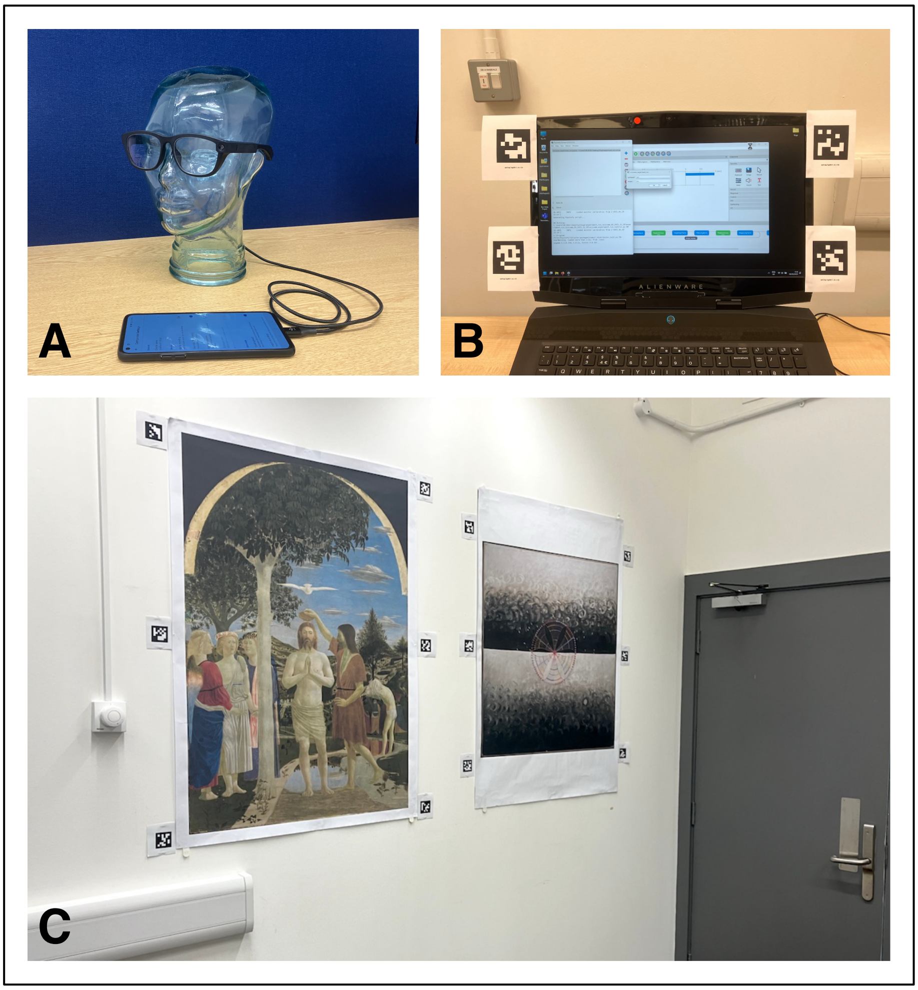

S3 Fig. Data recording setup.

Top-left (a): For gaze data collection, the mobile eye-tracker (Pupil Invisible) was attached to a companion recording device (OnePlus 8 Android phone) running the Pupil Invisible Companion App. Gaze data were collected at 200 Hz with a resolution of 192 × 192 pixels using two near-eye cameras with infrared illumination. Scene videos were recorded at 30 Hz with a resolution of 1088 × 1080 pixels, roughly corresponding to an 82° × 82° field of vision. **Top-right (b):** The laptop used (Dell Alienware 17 – 2019) displayed the maps and collected responses for the climate scale task and exit questionnaire. It featured a screen resolution of 1920 × 1080 pixels and a diagonal size of 17.3 inches (~44 cm), set in a 16:9 aspect ratio (~38 × 21.5 cm display dimensions). Although participants sat at a regular work desk, the distance from screen to participant varied and was not controlled; however, it is approximately noted that at an average distance of 57 cm, 1 cm on the screen roughly equates to 1 degree of visual angle. **Bottom (c):** Two artwork posters were displayed side by side in the lab, colour-printed on A0 papers (841 × 1189 mm) with a white border and surrounded by six April tags for positional referencing. For further technical details on April tags and their implementations in the Pupil Cloud, Pupil Cloud data streams, marker mapper and reference image mapper enrichments, see the following resources:
https://april.eecs.umich.edu/software/apriltag
https://docs.pupil-labs.com/core/software/pupil-capture/#markers
https://docs.pupil-labs.com/invisible/basic-concepts/data-streams/
https://docs.pupil-labs.com/enrichments/marker-mapper/
https://docs.pupil-labs.com/enrichments/reference-image-mapper/
