# Supplementary material for: Studying attention to IPCC climate change maps with mobile eye-tracking
Source: PLoS One. 2025 Jan 10;20(1):e0316909. doi: 10.1371/journal.pone.0316909 (PMC11723542; doi:10.1371/journal.pone.0316909)
Supplement: S4 Fig — (PDF) [file pone.0316909.s004.pdf]

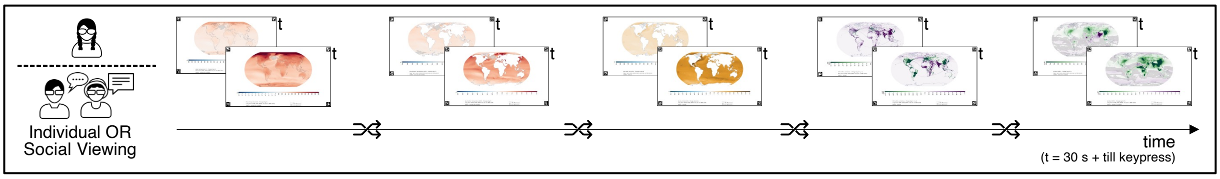

S4 Fig. Procedure diagram for viewing ten maps.

This diagram illustrates the time-course of viewing conditions for both individual and paired participants. Participants viewed the complete set of ten maps in a pairwise randomised order, starting with the near-future version followed by the far-future version of the same projection type. Each map was displayed for a minimum of 30 seconds, after which a small description appeared at the top, allowing participants to view the map as long as desired until proceeding to the next by pressing a spacebar key. Before any map presentation, a brief title screen (lasting approximately five seconds) introduced the content of the upcoming map with a couple of words and two icons (similar to those in Figure 1). In paired viewing conditions, participants were encouraged to discuss the maps if desired, although this varied among pairs. Each map remained visible until the group chose to proceed, provided at least 30 seconds had elapsed. The sequence of map presentations was randomised in pairs, with $N_{Stimulus} = 10$ as five pairs of near- and far-future projections. Note that a similar procedure was employed for viewing the two paintings (not illustrated here for succinctness), with participants deciding the order and duration of viewing for the printed posters.
