# Supplementary material for: Studying attention to IPCC climate change maps with mobile eye-tracking
Source: PLoS One. 2025 Jan 10;20(1):e0316909. doi: 10.1371/journal.pone.0316909 (PMC11723542; doi:10.1371/journal.pone.0316909)
Supplement: S5 Fig — (PDF) [file pone.0316909.s005.pdf]

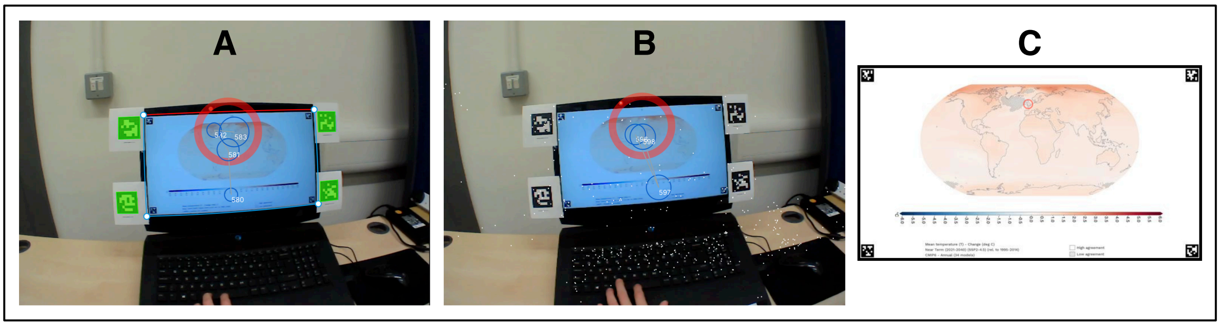

S5 Fig. Online data pre-processing using MME and RIM methods.

This figure demonstrates the two data processing methods used within Pupil Cloud for both screen-based (maps) and in-situ (paintings) stimuli. **Left (a):** The Marker Mapper Enrichment (MME) method involved placing four markers around the laptop screen (highlighted in green). The boundaries defined by these markers (shown with blue and red borders) depict the surface within which raw gaze data are normalised. However, this method was later abandoned due to relatively low data accuracy. **Middle (b):** The Reference Image Mapper (RIM) method required separate scanning recordings lasting about one minute and snapshot images for each stimulus. It employs a structure-from-motion technique to create a digital model from the video frames, shown as white dots superimposed on the scene camera view. The raw gaze data are then normalised against this model and the reference snapshot. Due to its relatively higher data accuracy, only data processed via RIM was used for analysis. **Right (c):** This panel shows the process of normalising raw gaze data against the model and reference image, where the red circle denotes the normalised location of the fixation with normalised XY coordinates relative to the pixels of the snapshot image. Note that the same preprocessing methods were applied to the supplementary painting stimuli (not shown here for brevity).
