# Supplementary material for: Studying attention to IPCC climate change maps with mobile eye-tracking
Source: PLoS One. 2025 Jan 10;20(1):e0316909. doi: 10.1371/journal.pone.0316909 (PMC11723542; doi:10.1371/journal.pone.0316909)
Supplement: S6 Fig — (PDF) [file pone.0316909.s006.pdf]

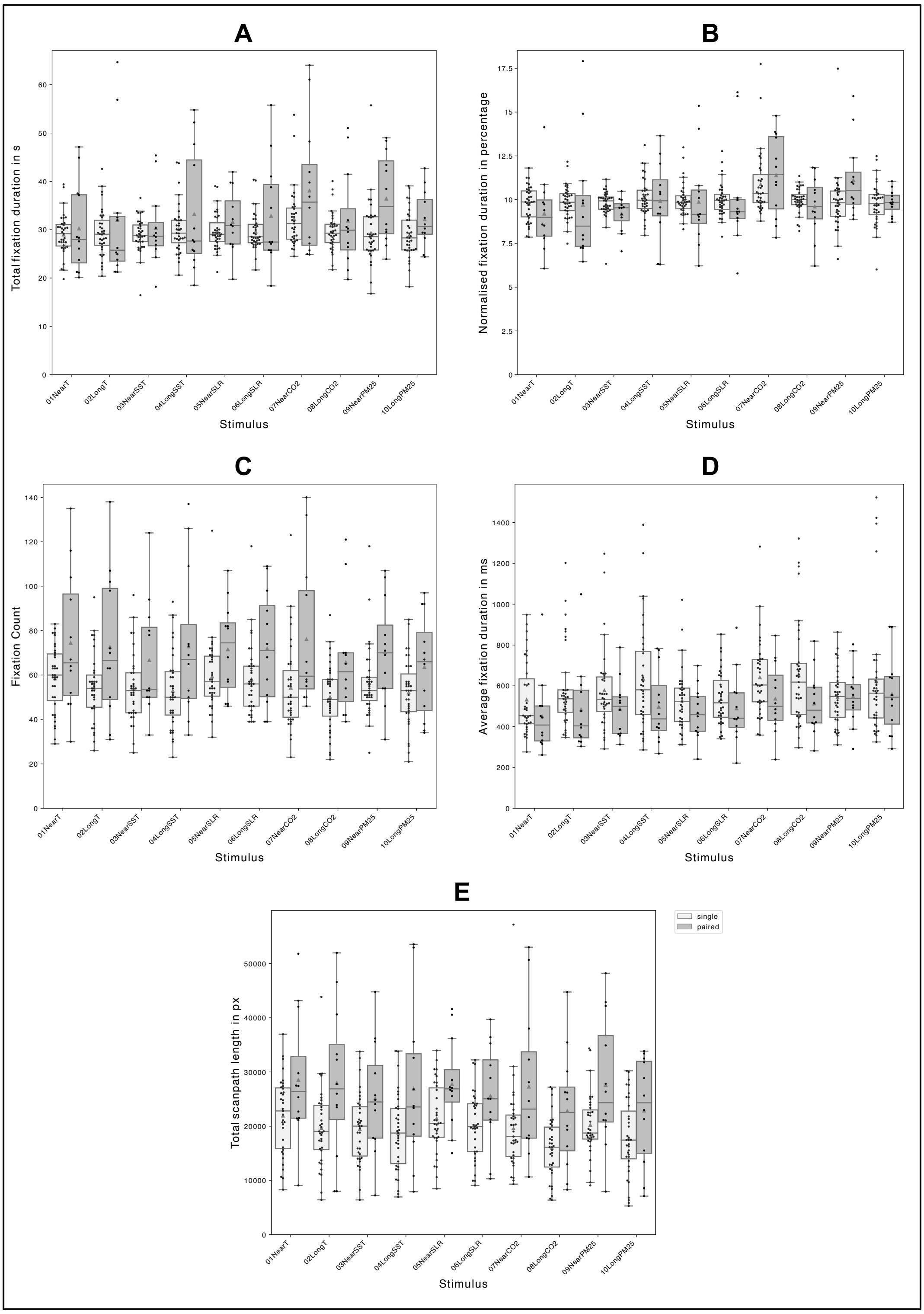

S6 Fig. Breakdown of box plots of descriptive statistics for main gaze metrics by map.

Supplementing the cumulative box plots, this figure details the breakdown of gaze metrics for ten individual maps. It follows the same format as the previous figure, presenting metrics for: **(a)** total fixation duration in milliseconds, **(b)** normalised fixation duration as a percentage (dwell time), **(c)** fixation count, **(d)** average fixation duration in milliseconds, and **(e)** total proxy scanpath length in pixels. Box plots are colour-coded in light and dark greys to represent single- ($N_{StimulusSingle} = 35$) and paired-viewing ($N_{StimulusPaired} = 12$) conditions, respectively. Each box plot shows the range (excluding outliers), IQR, median, and mean (marked with a small triangle). The categorical x-axis categorises the ten maps as different stimuli, while the y-axis denotes each metric. Scanpath distances are calculated similar to the previous figure.
