# Supplementary material for: Studying attention to IPCC climate change maps with mobile eye-tracking
Source: PLoS One. 2025 Jan 10;20(1):e0316909. doi: 10.1371/journal.pone.0316909 (PMC11723542; doi:10.1371/journal.pone.0316909)
Supplement: S7 Fig — (PDF) [file pone.0316909.s007.pdf]

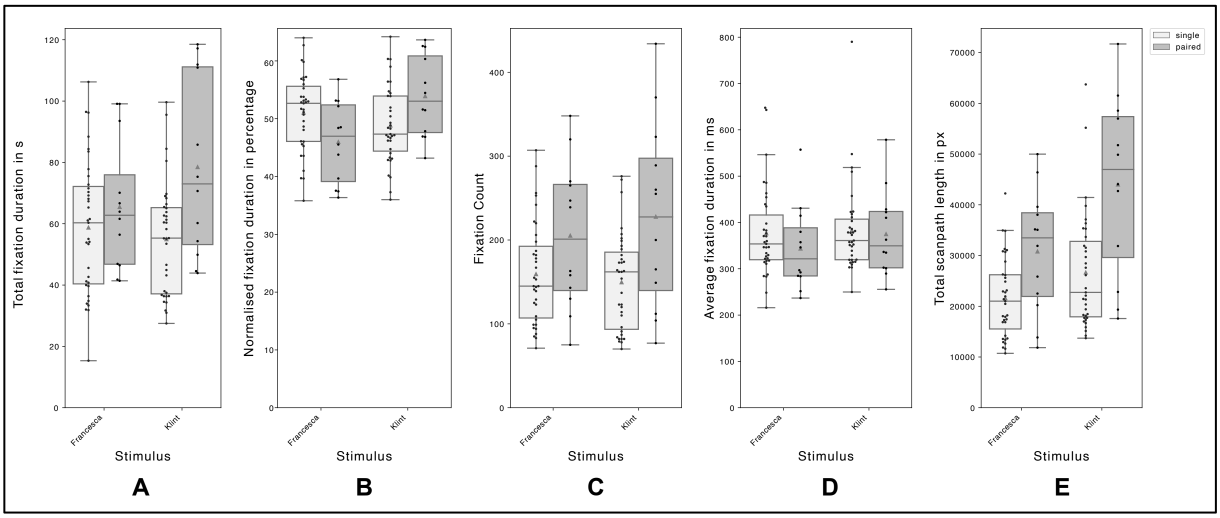

S7 Fig. Breakdown of box plots of descriptive statistics for main gaze metrics by painting.

This figure supplements the cumulative box plots by detailing the breakdown of gaze metrics for two individual paintings. It showcases the same five main gaze metrics: **(a)** total fixation duration in milliseconds (dwell time), **(b)** normalised fixation duration as a percentage, **(c)** fixation count, **(d)** average fixation duration in milliseconds, and **(e)** total proxy scanpath length in pixels. The box plots are colour-coded in light and dark greys to distinguish between single-viewing ($N_{SampleSingle} = 35$, $N_{StimulusSingle} = 35$) and paired-viewing ($N_{SamplePaired} = 12$, $N_{StimulusPaired} = 12$) conditions, as described in the legend at the top right. Each plot displays the range (excluding outliers), interquartile range (IQR), median, and mean (indicated by a small triangle overlay), providing a detailed view of the variability. Data points within each plot reflect the values of individual stimuli, hence these are the broken-down box plots corresponding to each painting. Note that the proxy scanpath distances, calculated for both viewing conditions, are the cumulative sums of the Euclidean distances between two consecutive fixations for each painting and participant. These distances are based on the arbitrary pixel values from the reference images used for each painting: 685 × 1000 pixels for The Baptism of Christ (Painting #1) and 983 × 1000 pixels for The Swan (Painting #2), which introduces some compatibility issues in unified analysis, and requires further normalisation. The categorical x-axis denotes the two different paintings as stimuli, while the y-axis denotes each gaze metric.
