# Supplementary material for: Studying attention to IPCC climate change maps with mobile eye-tracking
Source: PLoS One. 2025 Jan 10;20(1):e0316909. doi: 10.1371/journal.pone.0316909 (PMC11723542; doi:10.1371/journal.pone.0316909)
Supplement: S8 Fig — (PDF) [file pone.0316909.s008.pdf]

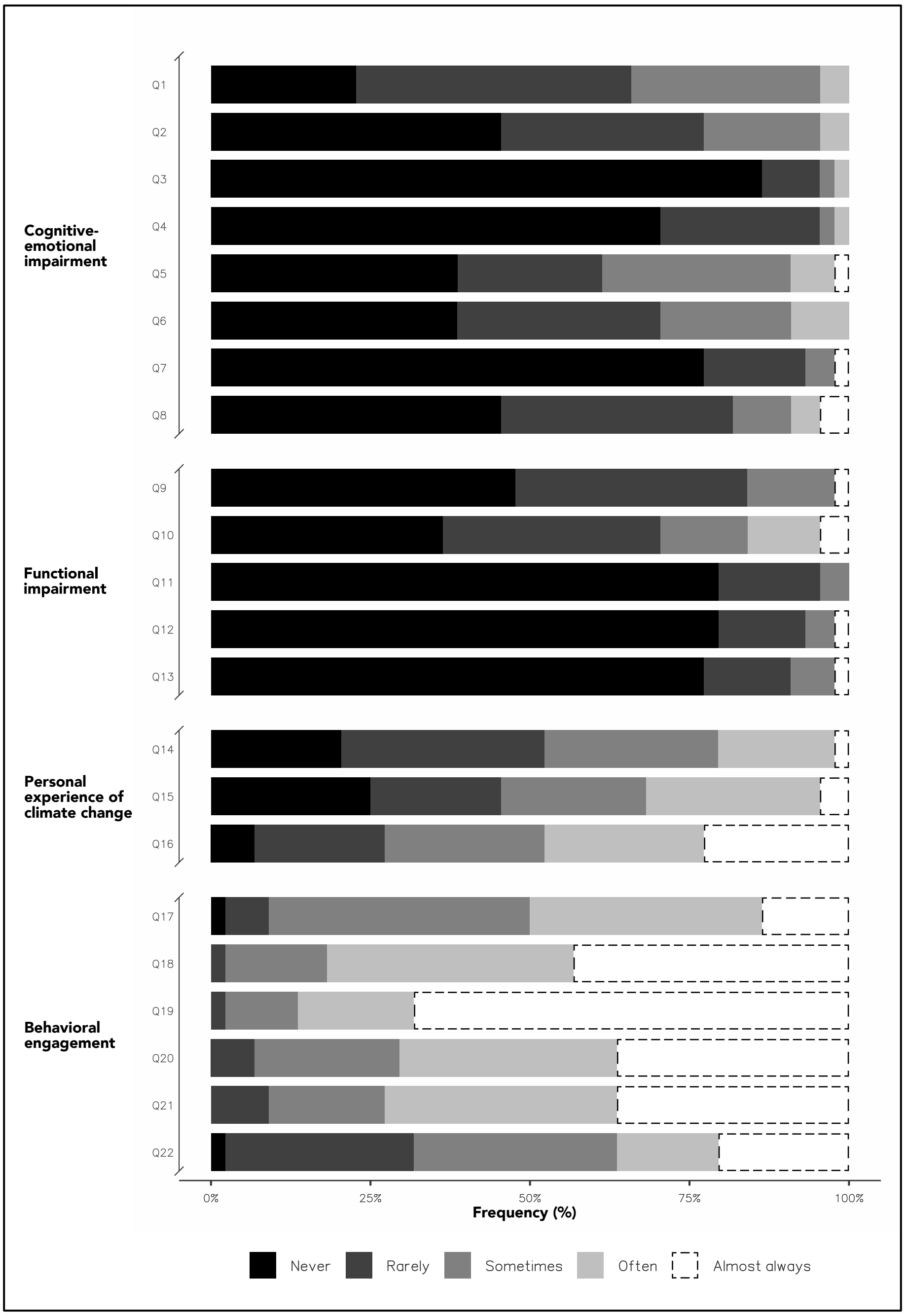

S8 Fig. Frequency plot of survey results, item-wise.

This frequency table presents the aggregated responses from the Climate Change Anxiety Scale (refer to Supplementary Material 1). The survey was conducted on a 5-point Likert scale across 22 items and presented here irrespective of single or paired viewing conditions. Responses are colour-coded from black to white, corresponding to the frequency from "never" to "almost always", as shown in the legend below the table. The scale includes four sub-scales, each associated with a set of questions: cognitive-emotional impairment (questions 1-8), functional impairment (questions 9-13), personal experience of climate change (questions 14-16), and behavioural engagement (questions 17-22). These sub-scales are labelled on the left side of the frequency plot. Note that $N_{Sample} = 44$, as responses from three participants were not recorded.
