# Supplementary material for: Studying attention to IPCC climate change maps with mobile eye-tracking
Source: PLoS One. 2025 Jan 10;20(1):e0316909. doi: 10.1371/journal.pone.0316909 (PMC11723542; doi:10.1371/journal.pone.0316909)
Supplement: S9 Fig — (PDF) [file pone.0316909.s009.pdf]

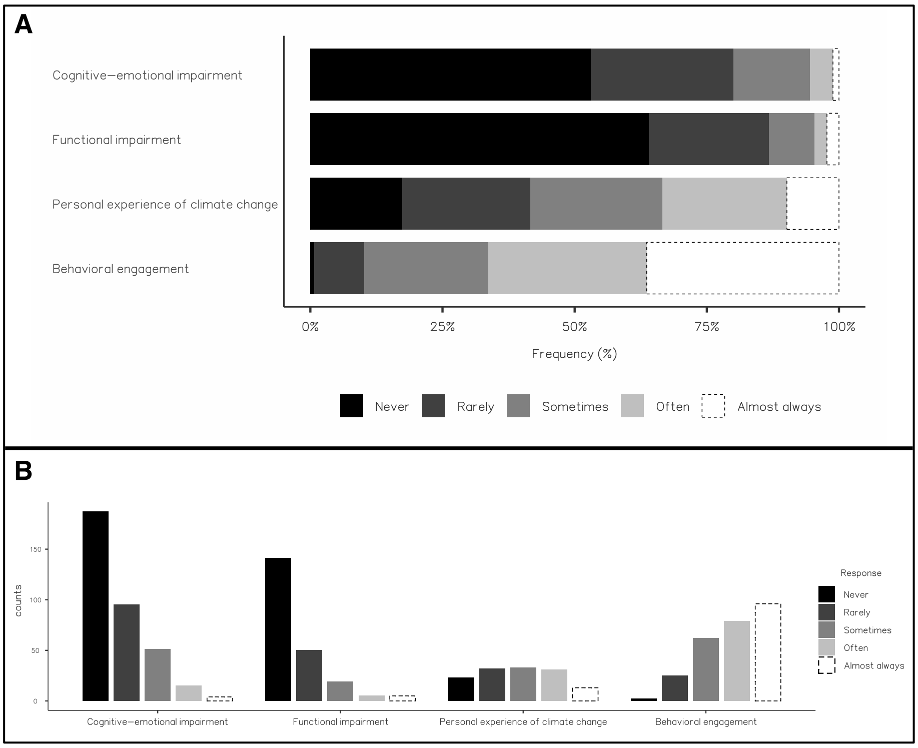

S9 Fig. Frequency plots of survey results, by four subcategories.

This figure displays the cumulative frequency table, aggregating responses into four sub-categories to show overall trends and frequency distributions within the Climate Change Anxiety Scale: (**a**) Four sub-categories plotted along a frequency X-axis, and (**b**) the same four subcategories visualised as count-based data, reflecting the differing number of questions per sub-scale. The y-axis counts reflect the total number of individual responses, varying by the number of questions in each sub-category: 8 questions under cognitive-emotional impairment, 5 under functional impairment, 3 under personal experience of climate change, and 6 under behavioural engagement. With N_{Sample} = 44 due to three unrecorded participant responses, the total N_{Count} for responses are: 353 for cognitive-emotional impairment, 220 for functional impairment, 225 for personal experience, and 264 for behavioural engagement. The 5-point Likert scale is colour-coded from black to white, as shown in the accompanying legends.
