# Supplementary material for: Studying attention to IPCC climate change maps with mobile eye-tracking
Source: PLoS One. 2025 Jan 10;20(1):e0316909. doi: 10.1371/journal.pone.0316909 (PMC11723542; doi:10.1371/journal.pone.0316909)
Supplement: S10 Fig — (PDF) [file pone.0316909.s010.pdf]

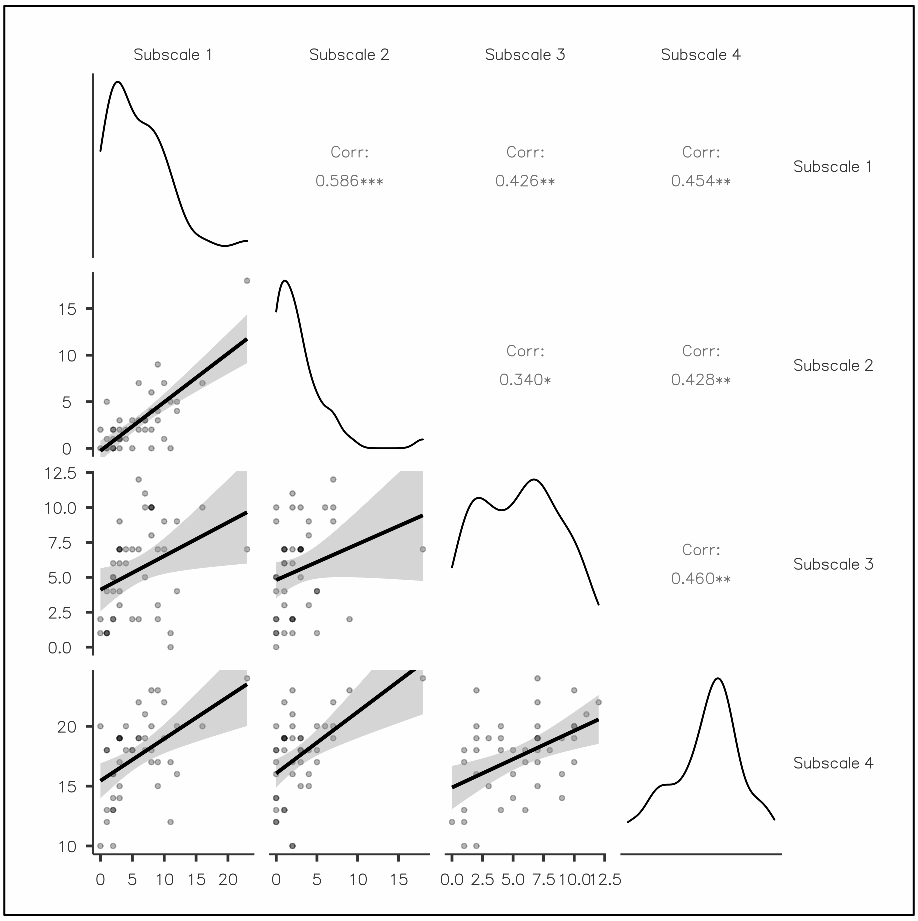

S10 Fig. Correlation plots between four subscales of the CCAS.

This figure presents the cross-correlation between the four subscales of the Climate Change Anxiety Scale (CCAS). Labels at the top and right (from Subscale 1 to Subscale 4) correspond to the four subscales of the CCAS. Along the diagonal axis from top-left to bottom-right, the graphs display the response distribution for each subscale, where 5-point Likert scale data for each question (treated as ordinal data) is converted to 0-4 scores. Below the diagonal, six correlation plots reveal all possible pairwise correlations among the subscales. Each plot includes individual data points, a linear correlation line, and confidence intervals for visual guidance. Above the diagonal, the corresponding areas display the Spearman's rho correlation coefficients for each pair of subscales.
Note that the sample size for these analyses was $N_{Sample} = 44$.
