# Supplementary material for: Studying attention to IPCC climate change maps with mobile eye-tracking
Source: PLoS One. 2025 Jan 10;20(1):e0316909. doi: 10.1371/journal.pone.0316909 (PMC11723542; doi:10.1371/journal.pone.0316909)
Supplement: S1 Table — (PDF) [file pone.0316909.s011.pdf]

Descriptives

	Condition	Total fixation duration in s	Normalised fixation duration in percentage	Fixation count	Average fixation duration in ms	Total scanpath length in px
N	single	350	350	350	350	350
	paired	120	120	120	120	120
Mean	single	29.7	10	55.12	586.43	19534.89
	paired	32.92	10	70.7	504.45	26288.61
Std. error mean	single	0.26	0.06	0.87	11.29	365.87
	paired	0.93	0.21	2.47	15.29	1036.95
Median	single	29.02	9.95	54	546.19	19280.35
	paired	29.45	9.68	65.5	479.26	25215.88
Standard deviation	single	4.81	1.2	16.2	211.18	6844.86
	paired	10.24	2.29	27.09	167.5	11359.19
Minimum	single	16.45	6.01	21	276.2	5285.11
	paired	18.19	5.78	30	221.36	7104.28
Maximum	single	55.73	17.75	125	1523	57201.18
	paired	64.65	17.91	140	1048.9	53560.01

S1 Table. Gaze metrics for maps.

This table presents descriptive statistics for five gaze metrics observed while viewing maps: total fixation duration (in seconds and as a percentage of total viewing time), fixation count, average fixation duration (in milliseconds), and total proxy scanpath length in pixels. The metrics are reported with the following statistics for each: observation size (N), mean (M), standard deviation (SD), standard error of the mean (SEM), minimum (Min), and maximum (Max) values. The data ($N_{MapStimulus}$ = 10) are segmented into single ($N_{SingleSample}$ = 35) and paired ($N_{PairedSample}$ = 12) viewing conditions, allowing for a direct comparison of these metrics under different social viewing contexts.
