# Supplementary material for: Studying attention to IPCC climate change maps with mobile eye-tracking
Source: PLoS One. 2025 Jan 10;20(1):e0316909. doi: 10.1371/journal.pone.0316909 (PMC11723542; doi:10.1371/journal.pone.0316909)
Supplement: S2 Table — (PDF) [file pone.0316909.s012.pdf]

Kruskal-Wallis

	χ^2	df	p	ε^2
Total fixation duration in s	1.7	1	0.193	0
Normalised fixation duration in percentage	3.22	1	0.073	0.01
Fixation count	28.69	1	<.001	0.06
Average fixation duration in ms	15.65	1	<.001	0.03
Total scanpath length in px	38.27	1	<.001	0.08

S2 Table. Nonparametric ANOVA (Kruskal-Wallis test) for maps, comparing single and paired viewing conditions.

This table details the results of a nonparametric ANOVA (Kruskal-Wallis test) used to analyse the differences in gaze metrics between single and paired viewing conditions for maps. Despite the exploratory nature of the research and the relatively low sample sizes (N = 35 for single and N = 12 for paired), significant differences were observed in fixation count, average fixation duration, and scanpath length, while no significant differences were found in total fixation duration. The table provides a detailed breakdown of the test results for each metric, including the chi-squared (χ^2) values, degrees of freedom (df), p-values, and effect sizes (ε^2). Note, although there is not a consensus on effect size interpretation, $\varepsilon^2 < .08$ may be assumed to be small, $\varepsilon^2 < .26$ assumed to be medium, $\varepsilon^2 >= .26$ assumed to be large.
