# Supplementary material for: Studying attention to IPCC climate change maps with mobile eye-tracking
Source: PLoS One. 2025 Jan 10;20(1):e0316909. doi: 10.1371/journal.pone.0316909 (PMC11723542; doi:10.1371/journal.pone.0316909)
Supplement: S3 Table — (PDF) [file pone.0316909.s013.pdf]

A - Descriptives

	Projection timeframe	Total fixation duration in s	Normalised fixation duration in percentage	Fixation count	Average fixation duration in ms	Total scanpath length in px
Mean	Near	30.74	10.08	60.51	548.73	22296.16
	Long	30.31	9.92	57.69	582.27	20222.32
Std. error mean	Near	0.44	0.11	1.35	11.31	561.84
	Long	0.44	0.1	1.35	14.98	570.83
Median	Near	29.15	9.88	56	519.62	21093.2
	Long	29	9.89	55	540.36	19544.69
Standard deviation	Near	6.78	1.64	20.65	173.35	8612.78
	Long	6.76	1.46	20.66	229.64	8750.73

B - Descriptives

	Projection type	Total fixation duration in s	Normalised fixation duration in percentage	Fixation count	Average fixation duration in ms	Total scanpath length in px
Mean	T	29.73	9.73	60.9	535.54	22749.26
	SST	30.13	9.85	57.8	578.21	20921.01
	SLR	30.32	10	62.15	519.15	22296.17
	CO2	31.98	10.45	57.09	617.88	19704.69
	PM25	30.47	9.97	57.55	576.73	20625.08
Std. error mean	T	0.73	0.17	2.24	20.16	969.22
	SST	0.67	0.13	2.22	22.92	924.52
	SLR	0.58	0.16	1.93	14.86	759.54
	CO2	0.8	0.17	2.33	22.09	963.33
	PM25	0.69	0.16	1.91	22.91	852.72
Median	T	28.41	9.8	58.5	487.4	22221.5
	SST	28.8	9.84	53	530.67	20033.92
	SLR	28.94	9.84	58	497.18	21146.47
	CO2	30.16	10.14	54.5	590.5	17778.25
	PM25	29.05	9.91	54.5	550.93	19390.62
Standard deviation	T	7.08	1.63	21.69	195.44	9396.89
	SST	6.49	1.22	21.51	222.26	8963.56
	SLR	5.58	1.59	18.68	144.12	7363.99
	CO2	7.77	1.66	22.63	214.19	9339.81
	PM25	6.65	1.56	18.5	222.12	8267.46

S3 Table. Gaze metrics for maps, broken down by projection timeframe and type.

This table extends the analysis of cumulative gaze metrics by breaking down the data into either two projection timeframes or five projection types. It presents the same five metrics using mean (M), median, standard deviation (SD), and standard error of the mean (SEMs) without distinguishing between single or paired viewing conditions for brevity. **Top (a)** As projection timeframe breakdown, metrics are categorised into short-term (2021-2040) and long-term (2081-2100) projections. The observation count for each category is based on five times the total sample size (N_{Sample} = 47), resulting in $N_{Observations}$ = 235 for each timeframe, as in each cell value. **Bottom (b)** As projection type breakdown, metrics are detailed for five types of projections: main temperature (T), sea surface temperature (SST), sea level rise (SLR), anthropogenic CO_2 emissions (CO2), and fine particulate matter $PM_{2.5}$ (PM25). The observation count for each type is based on twice the total sample size, resulting in $N_{Observations}$ = 94 for each type, as in each cell value.
