# Supplementary material for: Studying attention to IPCC climate change maps with mobile eye-tracking
Source: PLoS One. 2025 Jan 10;20(1):e0316909. doi: 10.1371/journal.pone.0316909 (PMC11723542; doi:10.1371/journal.pone.0316909)
Supplement: S4 Table — (PDF) [file pone.0316909.s014.pdf]

Descriptives

	Stimulus	Condition	Total fixation duration in s	Normalised fixation duration in percentage	Fixation count	Average fixation duration in ms	Total scanpath length in px
Mean	01NearT	single	29.01	9.77	58.6	533.67	21988.47
		paired	30.29	9.21	74.58	451.1	28576.68
	02LongT	single	29.44	9.88	54.46	583.67	19699.17
		paired	32.07	9.72	72.75	485.04	28036.92
	03NearSST	single	28.94	9.75	54.23	578.82	19499.82
		paired	30.37	9.19	66.83	488.31	25174.1
	04LongSST	single	30.16	10.13	53.06	635.75	18814.56
		paired	33.28	9.94	73	498.52	26956.82
	05NearSLR	single	29.64	10	59.43	529.42	21483.81
		paired	31.63	9.88	71.67	467.16	27790.66
	06LongSLR	single	29.66	10	58.23	536.5	20076.06
		paired	32.89	10.07	72	490.57	25646.38
	07NearCO2	single	32.25	10.88	54.34	642.27	19656.07
		paired	38.12	11.41	76.25	538.09	27327.28
	08LongCO2	single	29.63	9.97	50.26	656.27	16064.97
		paired	31.91	9.62	65.83	514.54	22839.75
	09NearPM25	single	29.36	9.85	56.34	544.9	20278.84
		paired	36.47	11.13	70.42	549.87	27619.02
	10LongPM25	single	28.95	9.75	52.26	623.06	17787.09
		paired	32.16	9.84	63.67	561.29	22918.46
Std. error mean	01NearT	single	0.72	0.17	2.51	30.23	1192.99
		paired	2.67	0.6	9.11	53.42	3393.83
	02LongT	single	0.83	0.16	2.6	34.24	1215.88
		paired	4.08	1	9.41	61.25	4035.72
	03NearSST	single	0.6	0.14	2.6	34.59	1080.85
		paired	2.25	0.28	7.45	40.56	2977.65
	04LongSST	single	0.87	0.19	2.91	44.05	1189.82
		paired	3.64	0.65	9.77	51.89	4216.38
	05NearSLR	single	0.64	0.16	2.72	25.55	1072.97
		paired	1.87	0.75	6	37.04	2382.96
	06LongSLR	single	0.66	0.16	2.7	22.14	1038.97
		paired	3.14	0.87	7.37	50.32	2677.83
	07NearCO2	single	1.03	0.31	3.34	31.45	1429.01
		paired	3.94	0.67	9.58	49.92	3969.73
	08LongCO2	single	0.66	0.12	2.54	43.24	938.7
		paired	2.93	0.49	7.46	44.31	3107.72
	09NearPM25	single	1.11	0.29	2.76	23.01	954.22
		paired	2.65	0.63	6.73	42.15	3486.96
	10LongPM25	single	0.81	0.21	2.53	51.74	1190.17
		paired	1.66	0.21	6.56	56.18	2815.41
Median	01NearT	single	29.18	9.87	60	454.16	22816.85
		paired	27.96	8.99	65.5	408.06	26378.1
	02LongT	single	29.06	9.85	54	536.45	19048.63
		paired	25.75	8.49	66.5	403.91	26891.85
	03NearSST	single	28.81	9.93	53	533.87	20015.09
		paired	28.63	9.54	53.5	499.67	24475.15
	04LongSST	single	29.28	9.97	50	580.53	18738.52
		paired	27.66	9.94	67	438.09	23553.26
	05NearSLR	single	29.04	9.85	57	522.46	20520.23
		paired	30.87	9.16	74.5	458.61	26892.28
	06LongSLR	single	28.54	9.96	56	516.82	19925.46
		paired	27.45	9.32	71	441.25	25065.54
	07NearCO2	single	31.28	10.35	50	604.08	18096.9
		paired	35.7	11.44	59.5	499.68	23160.18
	08LongCO2	single	29.27	10	49	618.23	16119.56
		paired	29.89	9.61	61.5	480.47	22533.31
	09NearPM25	single	28.56	9.82	53	547.81	18741.36
		paired	34.78	10.52	70	538.77	24338.31
	10LongPM25	single	28.3	9.77	53	564.06	17442.67
		paired	30.67	9.84	66	544.2	24325.92
Standard deviation	01NearT	single	4.24	1	14.87	178.84	7057.84
		paired	9.24	2.08	31.57	185.06	11756.56
	02LongT	single	4.91	0.93	15.38	202.59	7193.23
		paired	14.14	3.46	32.58	212.17	13980.13
	03NearSST	single	3.55	0.81	15.38	204.64	6394.38
		paired	7.78	0.98	25.8	140.5	10314.88
	04LongSST	single	5.16	1.09	17.21	260.58	7039.09
		paired	12.61	2.25	33.83	179.76	14605.98
	05NearSLR	single	3.77	0.95	16.1	151.14	6347.8
		paired	6.49	2.6	20.78	128.3	8254.83
	06LongSLR	single	3.89	0.96	15.99	130.97	6146.61
		paired	10.87	3	25.52	174.3	9276.27
	07NearCO2	single	6.09	1.83	19.75	186.05	8454.14
		paired	13.65	2.33	33.19	172.93	13751.54
	08LongCO2	single	3.93	0.69	15.01	255.79	5553.42
		paired	10.14	1.7	25.85	153.5	10765.45
	09NearPM25	single	6.54	1.69	16.34	136.11	5645.24
		paired	9.19	2.17	23.3	146.02	12079.2
	10LongPM25	single	4.79	1.24	14.98	306.07	7041.12
		paired	5.75	0.72	22.73	194.62	9752.87

S4 Table. Gaze metrics for maps, fully broken down.

Following the analysis presented in previous tables, this table further breaks down the same five gaze metrics by both viewing conditions (single and paired) and individual map stimuli (10 in total). To conserve space, only means and SEMs are reported: For the single viewing condition, each cell data is derived from a sample size of N_{Single} = 35. For the paired viewing condition, each cell data is based on a sample size of N_{Paired} = 12.
