# Supplementary material for: Studying attention to IPCC climate change maps with mobile eye-tracking
Source: PLoS One. 2025 Jan 10;20(1):e0316909. doi: 10.1371/journal.pone.0316909 (PMC11723542; doi:10.1371/journal.pone.0316909)
Supplement: S5 Table — (PDF) [file pone.0316909.s015.pdf]

AOI	Total fixation count (N = 47)	Total fixation duration in s (N = 47)	Relative dwell time in %
Top	64737	34806.87	80.87%
Bottom	18591	8232.07	19.13%

AOI	Condition	Fixation count (N_{Single} = 35, N_{Paired} = 12)	Total fixation duration in s (N_{Single} = 35, N_{Paired} = 12)	Relative dwell time in %
Top	Single	43959	24997.11	80.15%
Bottom	Single	13917	6191.21	19.85%
Top	Paired	20778	9809.76	82.78%
Bottom	Paired	4674	2040.85	17.22%

AOI	Condition	Map	Fixation count (N_{Single} = 35, N_{Paired} = 12)	Total fixation duration in s (N_{Single} = 35, N_{Paired} = 12)	Relative dwell time in %
Top	Single	01NearT	4422	2218.32	72.82%
Top	Single	02LongT	4395	2443.90	79.07%
Top	Single	03NearSST	4341	2478.18	81.57%
Top	Single	04LongSST	4413	2689.43	84.93%
Top	Single	05NearSLR	4650	2396.03	76.98%
Top	Single	06LongSLR	4725	2517.04	80.83%
Top	Single	07NearCO2	4116	2628.77	77.62%
Top	Single	08LongCO2	4254	2679.58	86.14%
Top	Single	09NearPM25	4239	2337.68	75.84%
Top	Single	10LongPM25	4404	2608.18	85.81%
Bottom	Single	01NearT	1731	828.10	27.18%
Bottom	Single	02LongT	1323	647.00	20.93%
Bottom	Single	03NearSST	1353	560.10	18.43%
Bottom	Single	04LongSST	1158	477.34	15.07%
Bottom	Single	05NearSLR	1590	716.57	23.02%
Bottom	Single	06LongSLR	1389	596.84	19.17%
Bottom	Single	07NearCO2	1590	757.79	22.38%
Bottom	Single	08LongCO2	1023	431.32	13.86%
Bottom	Single	09NearPM25	1677	744.90	24.16%
Bottom	Single	10LongPM25	1083	431.27	14.19%
Top	Paired	01NearT	2172	895.93	82.18%
Top	Paired	02LongT	2166	984.31	85.25%
Top	Paired	03NearSST	1905	868.74	79.45%
Top	Paired	04LongSST	2328	1068.20	89.16%
Top	Paired	05NearSLR	2016	874.97	76.84%
Top	Paired	06LongSLR	2262	1047.46	88.47%
Top	Paired	07NearCO2	2313	1187.84	86.55%
Top	Paired	08LongCO2	1914	929.20	80.89%
Top	Paired	09NearPM25	1935	1034.78	78.82%
Top	Paired	10LongPM25	1767	918.33	79.33%
Bottom	Paired	01NearT	513	194.26	17.82%
Bottom	Paired	02LongT	453	170.27	14.75%
Bottom	Paired	03NearSST	501	224.72	20.55%
Bottom	Paired	04LongSST	300	129.85	10.84%
Bottom	Paired	05NearSLR	564	263.75	23.16%
Bottom	Paired	06LongSLR	330	136.51	11.53%
Bottom	Paired	07NearCO2	432	184.67	13.45%
Bottom	Paired	08LongCO2	456	219.57	19.11%
Bottom	Paired	09NearPM25	600	278.01	21.18%
Bottom	Paired	10LongPM25	525	239.25	20.67%

S5 Table. Gaze metrics for maps divided into two AOIs.

This table serves as a supplementary, proof-of-concept exploration of fixation count, fixation duration (in seconds), and relative dwell time (in percentage) between two AOIs. The stimulus was divided into two parts as two AOIs: the top part covering the world map, and the bottom part covering the scale and its accompanying brief explanatory text. For stimuli displayed on a screen size of 1920 × 1080 pixels, the division line was set at the 767th horizontal pixel, marking the boundary between the map and scale areas. The table is organised into three sections: (a) Overall cumulative: At the top, presenting data aggregated across all viewing conditions and maps, showing how fixation metrics are distributed irrespective of specific conditions. (b) Divided by viewing conditions: In the middle, data are split between single and paired viewing conditions, highlighting differences in gaze behaviour between these contexts. (c) Fully broken down: At the bottom, data are detailed for each map and viewing condition individually, offering a granular look at interaction patterns. Across all metrics, an approximate 80-20% relative dwell time split was consistently observed between the upper (map) and lower (scale) parts of the stimulus.
