# Supplementary material for: Studying attention to IPCC climate change maps with mobile eye-tracking
Source: PLoS One. 2025 Jan 10;20(1):e0316909. doi: 10.1371/journal.pone.0316909 (PMC11723542; doi:10.1371/journal.pone.0316909)
Supplement: S6 Table — (PDF) [file pone.0316909.s016.pdf]

Descriptives

	Condition	Total fixation duration in s	Normalised fixation duration in percentage	Fixation count	Average fixation duration in ms	Scanpath length in px
N	single	70	70	70	70	70
	paired	24	24	24	24	24
Mean	single	56.97	0.50	154.87	380.51	24196.75
	paired	72.09	0.50	216.88	359.95	37468.70
Std. error mean	single	2.40	0.01	7.07	11.41	1205.88
	paired	5.30	0.02	20.27	18.68	3332.50
Median	single	56.78	0.50	153.50	357.63	21697.80
	paired	65.30	0.50	219.50	341.29	36599.86
Standard deviation	single	20.10	0.07	59.19	95.50	10089.12
	paired	25.97	0.08	99.29	91.53	16325.86
Minimum	single	15.34	0.36	70	216.00	10718.39
	paired	41.40	0.36	75	236.78	11845.43
Maximum	single	106.25	0.64	307	790.08	63731.17
	paired	118.51	0.64	434	578.53	71705.66

S6 Table. Gaze metrics for paintings.

This table presents descriptive statistics for five gaze metrics observed while viewing paintings: total fixation duration (in seconds and as a percentage of total viewing time), fixation count, average fixation duration (in milliseconds), and total proxy scanpath length in pixels. The metrics are reported with the following statistics for each: observation size (N), mean (M), standard deviation (SD), standard error of the mean (SEM), minimum (Min), and maximum (Max) values. The data ($N_{PaintingStimulus} = 2$) are segmented into single ($N_{SingleSample} = 35$) and paired ($N_{PairedSample} = 12$) viewing conditions, allowing for a direct comparison of these metrics under different social viewing contexts.
