# Supplementary material for: Studying attention to IPCC climate change maps with mobile eye-tracking
Source: PLoS One. 2025 Jan 10;20(1):e0316909. doi: 10.1371/journal.pone.0316909 (PMC11723542; doi:10.1371/journal.pone.0316909)
Supplement: S8 Table — (PDF) [file pone.0316909.s018.pdf]

Descriptives

	AOI	Condition	Total fixation duration in s	Fixation count	Scanpath length in px	Normalised fixation duration in percentage	Average fixation duration in ms
Mean	Francesca	paired	65.584	205.583	30868.837	0.460	344.332
		single	58.860	159.714	21671.617	0.511	377.368
	Klint	paired	78.597	228.167	44068.566	0.540	375.576
		single	55.072	150.029	26721.878	0.489	383.652
Std. error mean	Francesca	paired	6.165	25.222	3515.467	0.020	26.324
		single	3.693	10.344	1327.448	0.011	16.343
	Klint	paired	8.472	32.535	5115.004	0.020	26.877
		single	3.094	9.733	1940.344	0.011	16.159

S8 Table. Gaze metrics for paintings, fully broken down.

Following the analysis presented in previous tables, this table further breaks down the same five gaze metrics by both viewing conditions (single and paired) and individual painting stimuli (2 in total). To conserve space, only means and SEMs are reported: For the single viewing condition, each cell data is derived from a sample size of $N_{Single} = 35$. For the paired viewing condition, each cell data is based on a sample size of $N_{Paired} = 12$.
