# Supplementary material for: Studying attention to IPCC climate change maps with mobile eye-tracking
Source: PLoS One. 2025 Jan 10;20(1):e0316909. doi: 10.1371/journal.pone.0316909 (PMC11723542; doi:10.1371/journal.pone.0316909)
Supplement: S9 Table — (PDF) [file pone.0316909.s019.pdf]

Frequencies of Category

Category	Response	Counts
Cognitive-emotional impairment	Never	187
	Rarely	95
	Sometimes	51
	Often	15
	Almost always	4
Functional impairment	Never	141
	Rarely	50
	Sometimes	19
	Often	5
	Almost always	5
Personal experience of climate change	Never	23
	Rarely	32
	Sometimes	33
	Often	31
	Almost always	13
Behavioral engagement	Never	2
	Rarely	25
	Sometimes	62
	Often	79
	Almost always	96

S9 Table. Survey descriptive statistics by CCAS subscales.

This table displays the counts of responses on the Climate Change Anxiety Scale (CCAS), categorised using a 5-point Likert scale ranging from "never" to "almost always". The data are broken down into four subscales: cognitive-emotional impairment, functional impairment, personal experience of climate change, and behavioural engagement. Each subscale includes a different number of survey items, which results in varying total counts per subscale. Note that the sample size for this analysis is $N_{Sample} = 44$.
