# Supplementary material for: Studying attention to IPCC climate change maps with mobile eye-tracking
Source: PLoS One. 2025 Jan 10;20(1):e0316909. doi: 10.1371/journal.pone.0316909 (PMC11723542; doi:10.1371/journal.pone.0316909)
Supplement: S10 Table — (PDF) [file pone.0316909.s020.pdf]

Correlation Matrix

		Subscale 1	Subscale 2	Subscale 3	Subscale 4
Subscale 1	Spearman's rho	—			
	df	—			
	p-value	—			
	Kendall's Tau B	—			
	p-value	—			
Subscale 2	Spearman's rho	0.586	—		
	df	42	—		
	p-value	<.001	—		
	Kendall's Tau B	0.484	—		
	p-value	<.001	—		
Subscale 3	Spearman's rho	0.426	0.340	—	
	df	42	42	—	
	p-value	0.004	0.024	—	
	Kendall's Tau B	0.341	0.270	—	
	p-value	0.002	0.019	—	
Subscale 4	Spearman's rho	0.454	0.428	0.460	—
	df	42	42	42	—
	p-value	0.002	0.004	0.002	—
	Kendall's Tau B	0.355	0.330	0.352	—
	p-value	0.001	0.004	0.002	—

S10 Table. Survey correlation statistics for CCAS subscales.

This table presents the correlation statistics for the four subscales of the Climate Change Anxiety Scale (CCAS). Initially, responses on the 5-point Likert scale ranging from “never” to “almost always” were treated as ordinal data, with values from 0 to 4 assigned to each response. Subsequently, the sum of scores for each subscale was calculated. Correlations between the subscales were analysed using both Spearman’s rho and Kendall’s tau-b to assess the linear relationships. The table includes both correlation coefficients, significance levels (p-values), and degrees of freedom (df) for Spearman’s rho. These metrics provide insight into the cross-correlational dynamics among the subscales. The analysis, conducted with a relatively small sample size of $N_{Sample} = 44$ (irrespective of viewing conditions), consistently reveals positive linear relationships between the subscales, indicating a coherent pattern of responses across different dimensions of climate change anxiety.
