# Supplementary material for: Studying attention to IPCC climate change maps with mobile eye-tracking
Source: PLoS One. 2025 Jan 10;20(1):e0316909. doi: 10.1371/journal.pone.0316909 (PMC11723542; doi:10.1371/journal.pone.0316909)
Supplement: S11 Table — (PDF) [file pone.0316909.s021.pdf]

Correlation Matrix

		TOTAL Dwell time	TOTAL Fixation count	AVERAGE Fixation duration	AVERAGE Scanpath length
TOTAL CCAS	Spearman's rho	0.053	-0.037	0.03	0.014
	df	42	42	42	42
	p-value	0.731	0.81	0.848	0.927
	Kendall's Tau B	0.048	-0.012	0.02	0.007
	p-value	0.649	0.911	0.847	0.943

S11 Table. Correlations between gaze metrics and total CCAS score.

This table extends the analysis from the previous correlation table by examining the relationship between the total Climate Change Anxiety Scale (CCAS) score (treating it again as ordinal data) and four gaze metrics (as continuous data). The total CCAS score was calculated for each participant based on the cumulative sum of their responses on the 5-point Likert scale, based on the high positive linear correlation between its four subscales. The gaze metrics reported here include total dwell time, total fixation count, average fixation duration, and average proxy scanpath length. Correlation coefficients (Spearman's rho and Kendall's tau-b) along with p-values and degrees of freedom (df) are presented to assess the statistical significance of these relationships. No significant correlation was found between the total CCAS score and any of the gaze metrics, indicating that there is no apparent relationship between these relatively raw gaze metrics and self-reported anxiety scores as measured by the CCAS. Note that $N_{Sample} = 44$.
