# Supplementary material for: Studying attention to IPCC climate change maps with mobile eye-tracking
Source: PLoS One. 2025 Jan 10;20(1):e0316909. doi: 10.1371/journal.pone.0316909 (PMC11723542; doi:10.1371/journal.pone.0316909)
Supplement: S1 File — (PDF) [file pone.0316909.s022.pdf]

S1 File. The Climate Change Anxiety Scale (CCAS).

From: Clayton, S., & Karazsia, B. T. (2020). Development and validation of a measure of climate change anxiety. Journal of Environmental Psychology, 69, 101434. https://doi.org/10.1016/j.jenvp.2020.101434
The Climate Change Anxiety Scale is a 22-item measure of the emotional response to climate change. The measure has four sub-scales including cognitive and emotional impairment, functional impairment, personal experience of climate change, and behavioral engagement. The scale has been validated in the U.S.

ITEMS:

Please rate how often the following statements are true of you.

Cognitive-emotional impairment (Subscale 1)

1. Thinking about climate change makes it difficult for me to concentrate.
2. Thinking about climate change makes it difficult for me to sleep.
3. I have nightmares about climate change.
4. I find myself crying because of climate change.
5. I think, "why can't I handle climate change better?"
6. I go away by myself and think about why I feel this way about climate change.
7. I write down my thoughts about climate change and analyze them.
8. I think, "why do I react to climate change this way?"

Functional impairment (Subscale 2)

9. My concerns about climate change make it hard for me to have fun with my family or friends.
10. I have problems balancing my concerns about sustainability with the needs of my family.
11. My concerns about climate change interfere with my ability to get work or school assignments done.
12. My concerns about climate change undermine my ability to work to my potential.
13. My friends say I think about climate change too much.

Personal experience of climate change (Subscale 3)

14. I have been directly affected by climate change.
15. I know someone who has been directly affected by climate change.
16. I have noticed a change in a place that is important to me due to climate change.

Behavioral engagement (Subscale 4)

17. I wish I behaved more sustainably.
18. I recycle.
19. I turn off lights.
20. I try to reduce my behaviors that contribute to climate change.
21. I feel guilty if I waste energy.
22. I believe I can do something to help address the problem of climate change.

Response Options:

Never - 1
Rarely - 2
Sometimes - 3
Often - 4
Almost always – 5
